# Chemerin enhances mesenchymal features of glioblastoma by establishing autocrine and paracrine networks in a CMKLR1-dependent manner

**DOI:** 10.1038/s41388-022-02295-w

**Published:** 2022-04-22

**Authors:** Jianqi Wu, Shuai Shen, Tianqi Liu, Xiufang Ren, Chen Zhu, Qingyu Liang, Xiao Cui, Ling Chen, Peng Cheng, Wen Cheng, Anhua Wu

**Affiliations:** 1 Department of Neurosurgery, The First Hospital of China Medical University, Shenyang, China; 2 Departement of Pathology, Shengjing Hospital of China Medical University, Shenyang, China; 3 Department of Neurosurgery, Chinese People’s Liberation Army of China (PLA) General Hospital, Medical School of Chinese PLA, Institute of Neurosurgery of Chinese PLA, Beijing, China

**Keywords:** CNS cancer, Oncogenes, Prognostic markers

## Abstract

Glioblastoma multiforme (GBM) with mesenchymal features exhibits enhanced chemotherapeutic resistance and results in reduced overall survival. Recent studies have suggested that there is a positive correlation between the GBM mesenchymal status and immune cell infiltration. However, the mechanisms by which GBM acquires its mesenchymal features in a tumor immune microenvironment-dependent manner remains unknown. Here, we uncovered a chemerin-mediated autocrine and paracrine network by which the mesenchymal phenotype of GBM cells is strengthened. We identified chemerin as a prognostic secretory protein mediating the mesenchymal phenotype-promoting network between tumor-associated macrophages (TAMs) and tumor cells in GBM. Mechanistically, chemerin promoted the mesenchymal features of GBM by suppressing the ubiquitin-proteasomal degradation of CMKLR1, a chemerin receptor predominantly expressed on TAMs and partially expressed on GBM cells, thereby enhancing NF-κB pathway activation. Moreover, chemerin was found to be involved in the recruitment of TAMs in the GBM tumor microenvironment. We revealed that chemerin also enhances the mesenchymal phenotype-promoting ability of TAMs and promotes their M2 polarization via a CMKLR1/NF-κB axis, which further exacerbates the mesenchymal features of GBM. Blocking the chemerin/CMKLR1 axis with 2-(α-naphthoyl) ethyltrimethylammonium iodide disrupted the mesenchymal network and suppressed tumor growth in GBM. These results suggest the therapeutic potential of targeting the chemerin/CMKLR1 axis to block the mesenchymal network in GBM.

## Introduction

Glioblastoma (GBM) is the most common and aggressive primary brain tumor, and there is an increasing urgency to develop more effective therapies for this devastating disease [1]. Recent genomic characterization of GBM has provided a foundation to elucidate the associated molecular mechanisms, establish precise diagnostic tools, and develop promising therapeutic regimens for GBM. GBM with mesenchymal features (mesenchymal GBM) is characterized by the increased expression of mesenchymal markers (CD44, Vimentin (VIM), N-Cadherin (N-Ca), and ALDH1A3) and a more complex immune-suppressive environment [1]. These patients need more intensive therapeutics and suffer from a high recurrent rate, according to either Verhaak’s [1, 2] or Phillips’s [3] classification scheme. The enhancement of mesenchymal features is observed during the initiation, progression, and recurrence of GBM and is believed to play an important role in facilitating GBM cell invasion and resistance to therapies [4]. Therefore, clarifying the mechanisms underlying the enrichment of mesenchymal features may provide valuable clues for treating GBM.

Previously, we and other researchers found that mesenchymal GBMs could be characterized by abundant immune cell infiltration and that tumor-associated macrophages (TAMs) are the dominant non-tumor cells facilitating GBM progression [1, 2, 5–7]. Indeed, TAM abundance is substantially associated with the mesenchymal subtype and enhances the mesenchymal features of GBM cells [7, 8]. Furthermore, mesenchymal GBM cells have been shown to promote TAM recruitment [9, 10]. These observations suggest that there may exist a TAM-dependent intercellular network driving the enhancement of mesenchymal features in GBM. However, the mechanisms by which GBM cells activate the mesenchymal phenotype-promoting effects of TAMs remain elusive.

Chemerin, also known as retinoic acid receptor responder protein 2 (RARRES2), is a secreted protein that serves as a ligand for the G protein-coupled receptor chemokine-like receptor 1 (CMKLR1 or ChemR23) and other two poorly studied receptors, namely G protein-coupled receptor-1 (GPR1) and chemokine receptor-like 2 (CCRL2) [11]. Increasing studies have suggested that chemerin contributes to the regulation of multiple pathophysiological processes including tissue inflammation and glucose homeostasis. Notably, it also serves as a condition-dependent pro-tumor or anti-tumor factor in tumorigenesis [12]. Moreover, as an important chemokine, chemerin is well known for its potent innate immune cell-recruiting ability [13–15]. This could indicate its potential role in tumor microenvironment (TME) construction [13, 15–17]. To our knowledge, it remains unclear whether chemerin could mediate tumor progression and influence the TME in GBM. Therefore, this study aimed to elucidate the molecular mechanisms regulating mesenchymal features enhancement in the GBM TME. We further identified a novel mechanism underlying a mesenchymal phenotype-promoting network between GBM cells and TAMs, which highlights the chemerin/CMKLR1 axis as a promising target for GBM therapy.

## Results

### Tumor-derived chemerin is a key factor that is positively associated with the GBM mesenchymal phenotype

**Fig. 1:**
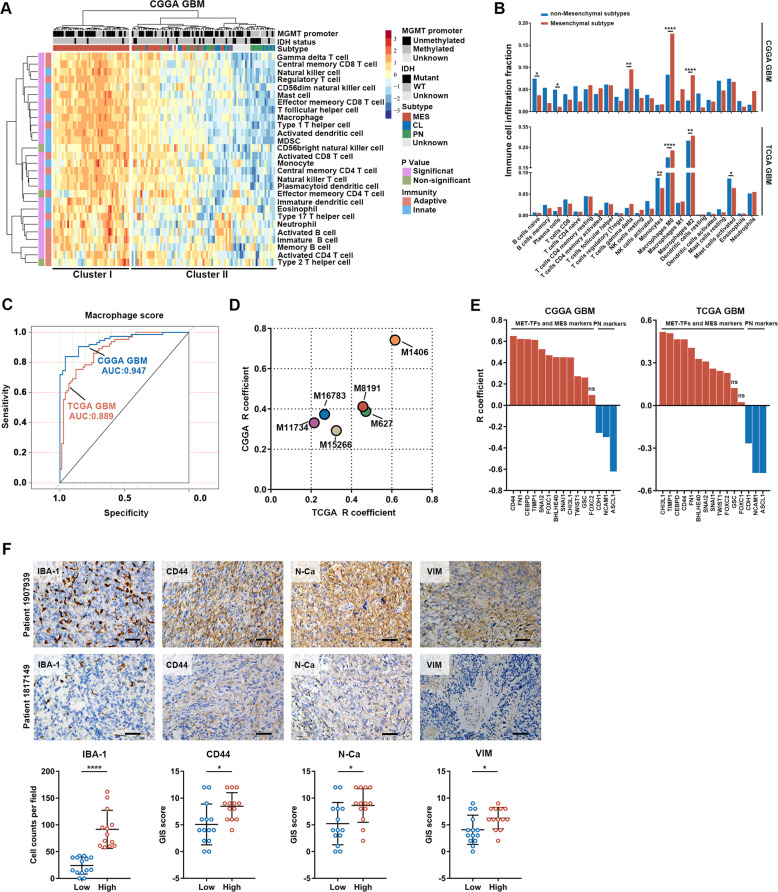
TAMs are positively correlated with mesenchymal status in GBM. **A** Heat map showing 28 immune cell-associated mRNA signature expression patterns between two classified GBM subtypes (Cluster I and Cluster II). **B** Immune cell infiltration fractions (*n* = 22) were compared between mesenchymal and non-mesenchymal GBM. **C** Sensitivity and specificity of macrophage ssGSEA score on diagnosing mesenchymal GBM. **D** The scatterplot showing R coefficients of Pearson’s correlation between mesenchymal related signature scores and macrophage scores. **E** Column diagram showing R coefficients of Pearson’s correlation between macrophage score and expression of indicated markers. MES mesenchymal, PN proneural. **F** Representative IHC images and staining quantification of indicated markers in patients’ samples with low and high TAM infiltration. Scale bars: 50 μm. Low TAMs infiltration group, *n* = 14; High TAM infiltration group, *n* = 13. Data is presented as means ± SD. Statistical significance in (**A**), (**B**), and (**F**) was analyzed using Student’s *t* test. ns not significant, *p < 0.05, **p < 0.01, ****p < 0.0001.

To compare the characteristics of the immune microenvironment among GBM subtypes, transcriptomic data of patients with GBM from the CGGA database were analyzed. The TCGA cohort was additionally analyzed for validation. Unsupervised clustering based on immune cell frequencies showed that mesenchymal GBM was significantly enriched in Cluster I (chi-square test, *P* < 0.0001), which was characterized by the enrichment of multiple immune cell ssGSEA (single sample GSEA) scores [18] (Fig. 1A; Supplementary Fig. S1A). TAM score (mostly M0- and M2-subtype macrophages) was more frequently enriched in mesenchymal GBMs than non-mesenchymal GBMs (Fig. 1B; Supplementary Fig. S1B). Assessment of the GSVA (gene set variation analysis) score showed that macrophage abundance was positively associated with mesenchymal GBM (Fig. 1C), the mesenchymal ssGSEA score (Fig. 1D; Supplementary Table S2), mesenchymal phenotype-related transcription factors (MES-TFs), and mesenchymal markers (Fig. 1E; Supplementary Fig. S1C, D). Moreover, histological analysis of GBM tissues verified that greater IBA-1^+^ TAM infiltration was related to enhanced mesenchymal features (increased CD44, N-Ca, and VIM expression; Fig. 1F).

**Fig. 2:**
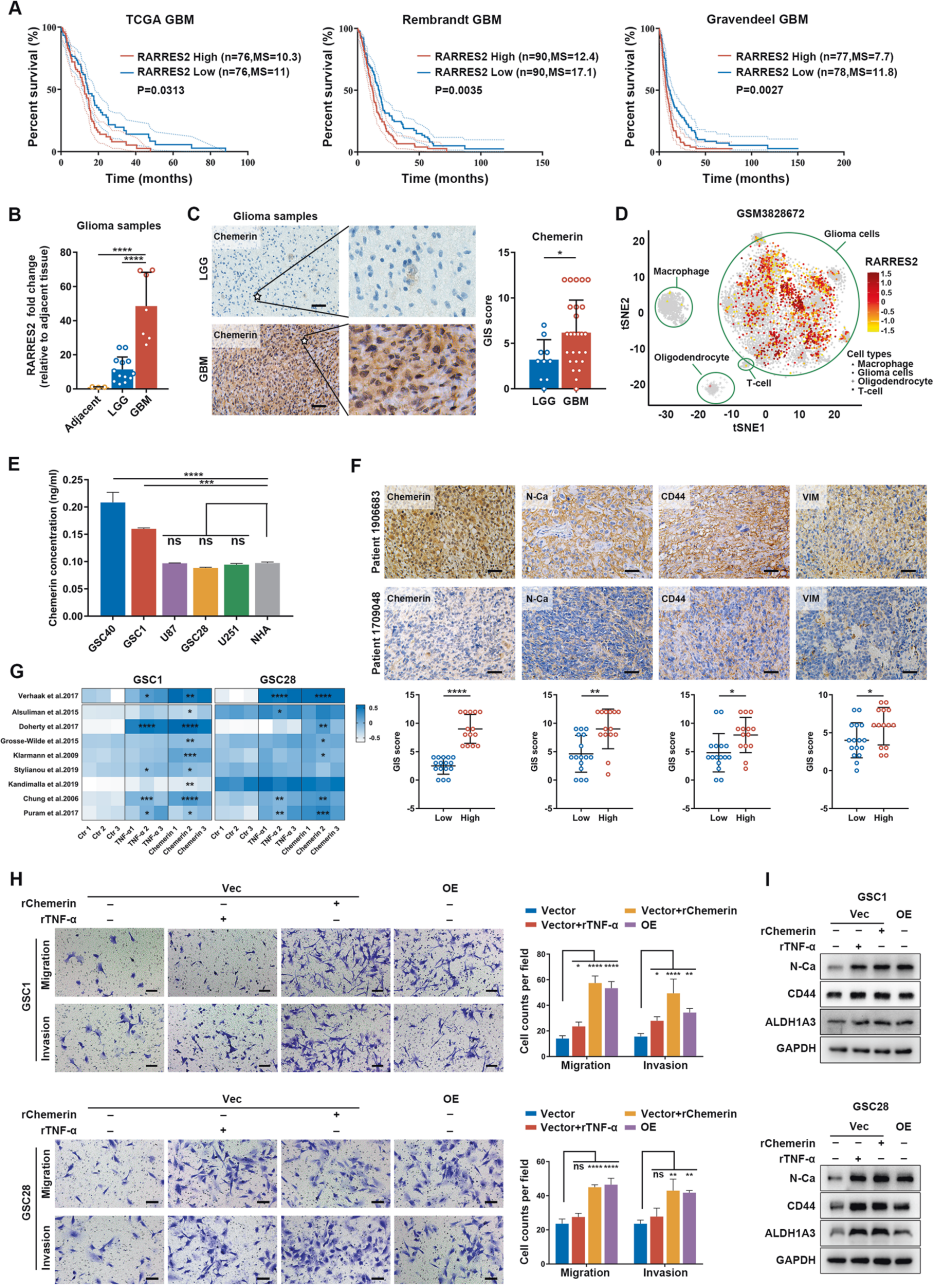
Chemerin is a GBM cell-derived prognostic factor that promotes the mesenchymal features of GBM. **A** Kaplan–Meier survival analysis of GBM based on high (> median level) or low (< median level) *RARRES2* expression levels. MS: median survival. *n* = 152 in TCGA cohort; *n* = 180 in Rembrandt cohort; *n* = 155 in Gravendeel cohort. **B** Real-time PCR analysis of *RARRES2* in clinical tissue samples. Adjacent sample, *n* = 3; LGG sample, *n* = 13; GBM sample, *n* = 8. **C** Representative IHC images and staining quantification of chemerin expression in glioma samples. Scale bars: 50 μm. LGG sample, *n* = 10; GBM sample, *n* = 28. **D** t-distributed stochastic neighbor embedding (tSNE) plot of *RARRES2* in GSM3828672 single-cell RNA sequencing dataset. **E** Enzyme-linked immunosorbent assay of chemerin expression in the supernatant from GBM cells and normal human astrocyte (NHA). *n* = 3. **F** Representative IHC images and staining quantification of indicated mesenchymal markers in GBM tissues with low and high chemerin expression. Scale bars: 50 μm. Low chemerin expression group, *n* = 16; High chemerin group, *n* = 14. **G** Heatmap of Verhaak mesenchymal subtype identification scores and mesenchymal GSVA scores of indicated rTNF-α or rChemerin treated GSCs. *n* = 3. **H** Representative images and cell count quantification of the migration and invasion analysis of rTNF-α treated, rChemerin treated, or chemerin overexpressed GSCs. *n* = 3. **I** Western blotting analysis of mesenchymal markers in indicated rTNF-α treated, rChemerin treated, or chemerin overexpressed GSCs. Data is presented as means ± SD. Differences in survival were analyzed using log-rank tests. Statistical significance in (**B**), (**C**), and (**F**) was analyzed using Student’s *t* test. Statistical significance in (**E**), (**G**), and (**H**) was analyzed using one-way ANOVA analyses. ns not significant, *p < 0.05, **p < 0.01, ***p < 0.001, ****p < 0.0001.

Next, to identify key factors associated with both mesenchymal status and TAM infiltration, we performed the analysis described in the Supplementary Information. *RARRES2*, which encodes chemerin, was finally selected for further investigation (Supplementary Table S3). We found that patient with GBM with reduced overall survival tended to have higher chemerin expression both in tumor sites and serum (Fig. 2A to C; Supplementary Fig. S2A, B). Moreover, although *RARRES2* elevation was positively related to a Chr.7 amplification event possibly owing to its origin gene site in Chr.7 (Supplementary Fig. S2C), the *RARRES2* expression level was still a prognostic factor independent of the Chr.7 gain event in GBM patients, as indicated by multivariate Cox analysis (Supplementary Fig. S2D). Single cell RNA-seq data of GBM tissue verified that *RARRES2* was mainly expressed in malignant cell populations (Fig. 2D) [19]. This was further verified in patient-derived GSCs, comparing them with normal human astrocytes (Fig. 2E).

Subsequently, we found that elevated *RARRES2* in patients with GBM implied an increased likelihood of having mesenchymal, IDH-wild-type, and unmethylated MGMT promoter tumors (Supplementary Table S4). A valuation of GBM tissues showed that the expression of mesenchymal markers (N-Ca, CD44, and VIM) was positively correlated with higher chemerin expression (Fig. 2F). The identification of the highest *RARRES2* expression in the mesenchymal phenotype also suggested a tight connection between chemerin and mesenchymal GBM (Supplementary Fig. S2E). Furthermore, single cell RNA-seq data of GBMs showed that *RARRES2* expression was significantly upregulated in GBM cell subpopulations with higher mesenchymal scores (MES1 and MES2, Supplementary Fig. S2F) [19]. Similarly, RNA sequencing and enzyme-linked immunosorbent assay of GSCs showed that chemerin expression was higher in GSCs with enhanced mesenchymal features, despite mesenchymal U87MG cells presenting negligible chemerin expression (Fig. 2E; Supplementary Fig. S2G) [20, 21]. Considering the tight connection between chemerin expression and lipid metabolism [22] and exaggerated lipogenesis and lipid metabolism found in gliomas [23], we further evaluated the lipid metabolism status of mesenchymal GBM cells (GSC1, GSC40, and U87MG) using lipid metabolism-related GSVA scores. Results showed that the lipid metabolism status was relatively higher in GSC1 and GSC40 cells as compared with that in U87MG cells (Supplementary Fig. S2H; Supplementary Table S5). This could be a possible explanation for the diversity of chemerin expression regardless of the GBM cell molecular phenotype. Taken together, these findings suggest that chemerin is a malignant factor associated with mesenchymal GBM.

### Chemerin enhances the mesenchymal features of GBM cells in an autocrine manner

To examine the effects of chemerin on the regulation of mesenchymal features in GBM cells, we conducted RNA sequencing on recombinant chemerin (rChemerin)-treated GSCs. The data showed that rChemerin-treated GSCs had higher Verhaak mesenchymal scores and several mesenchymal related GSVA scores with reference to GSCs treated with recombinant TNF-α (rTNF-α), a potent mesenchymal phenotype-promoting factor in GBM [24–26] (Fig. 2G). Interestingly, rChemerin treatment did not change the mesenchymal status of GSC40 cells, which already had abundant mesenchymal features and chemerin expression (Supplementary Fig. S2G), highlighting the limitation of its pro-mesenchymal effect on high chemerin-expressing GBM cells with high mesenchymal features (Supplementary Fig. S3A, B). Thus, to further explore the effect of chemerin on mesenchymal features of GBM, we overexpressed it in GSC1 and GSC28 cells and knocked it down in GSC1 and GSC40 cells (Supplementary Fig. S2G; Supplementary Fig. S3C, D). Similar to that with rTNF-α treatment, chemerin overexpression or rChemerin treatment significantly enhanced the migratory and invasive abilities of GSCs (Fig. 2H). Upregulated expression of well-established MES-TFs and mesenchymal markers was also observed in rChemerin-treated and chemerin-overexpressing GSCs (Fig. 2I; Supplementary Fig. S4A). In contrast, chemerin knockdown significantly suppressed the migratory and invasive abilities of GSCs and downregulated the expression of MES-TFs and mesenchymal markers, whereas rChemerin treatment reversed these mobility-inhibitory effects (Supplementary Fig. S4B to D). Together, these results suggest a potent enhancing effect of chemerin on the mesenchymal features of GBM cells.

### CMKLR1 is indispensable for activating chemerin signaling in GBM cells

Previous studies have suggested that CMKLR1 is the main receptor for chemerin in various cell types [12]. Similar to that of *RARRES2*, *CMKLR1* expression was elevated in mesenchymal GBM compared with expression in the other two subtypes (Supplementary Fig. S5A). Although *CMKLR1* alone only had prognostic value in the Rembrandt GBM dataset (Supplementary Fig. S5B), it could strengthen the prognostic implications of *RARRES2* for patients with GBM. We found that higher *RARRES2* expression indicated poor outcomes in patients with high *CMKLR1* expression, but it had limited prognostic implications in patients with low *CMKLR1* expression (Supplementary Fig. S5C). Moreover, the patient group with high *CMKLR1* and *RARRES2* expression showed a significantly higher frequency of mesenchymal GBM (Supplementary Table S6), suggesting a tight correlation between the expression of the chemerin/CMKLR1 axis and the mesenchymal phenotype in GBM.

To investigate whether CMKLR1 was the only receptor that could elicit the effect of chemerin in GBM cells, we knocked down three verified receptors of chemerin, GPR1, CMKLR2, and CMKLR1, in chemerin-overexpressing GSCs [27]. Results showed that only CMKLR1 knockdown suppressed the upregulation of mesenchymal markers in chemerin-overexpressing GSCs (Supplementary Fig. S6A to C). Moreover, CMKLR1 knockdown had a similar inhibitory effect as chemerin neutralization and treatment with α-NETA, a CMKLR1 inhibitor, on invasion/migration and mesenchymal marker expression in chemerin-overexpressing GSCs (Supplementary Fig. S7A, B) [28]. Together, these results suggest that CMKLR1 plays an important role in chemerin-mediated enhancement of mesenchymal features in GBM cells.

### Chemerin inhibits ubiquitin-proteasomal degradation of CMKLR1 in GBM cells

**Fig. 3:**
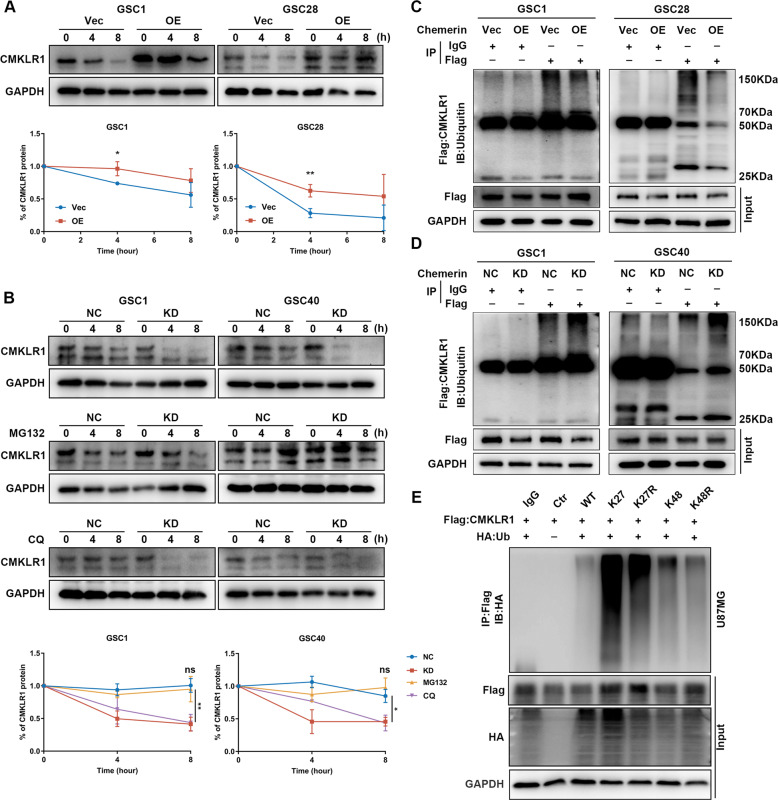
Chemerin suppresses K27/K48 ubiquitin chain of CMKLR1 in GBM cell. **A** Representative western blotting images and relative gray value quantification of time-dependent CMKLR1 expression change in indicated chemerin overexpressed GSCs after CHX treatment. *n* = 3. **B** Representative western blotting images and relative gray value quantification of time-dependent CMKLR1 expression change in indicated chemerin knockdown GSCs after CHX treatment, along with or without MG132 or Chloroquine (CQ) treatment. *n* = 3. **C**, **D** The pulldown assay of exogenous Flag-tagged CMKLR1 in indicated chemerin overexpressed or knockdown GSCs. The protein levels of ubiquitin were evaluated by western blotting. **E** The pulldown assay of exogenous Flag-tagged CMKLR1 and HA-tagged ubiquitin (WT, K27 only, K27R mutant, K48 only, K48R mutant) in U87MG cells. The protein levels of HA were evaluated by western blotting. Data is presented as means ± SD. Statistical significance in (**A**) and (**B**) was analyzed using Student’s *t* test. ns not significant, *p < 0.05, **p < 0.01.

Interestingly, although chemerin overexpression did not significantly affect *CMKLR1* mRNA levels, CMKLR1 protein expression was increased in chemerin-overexpressing GSCs and decreased in chemerin-knockdown GBMs (Supplementary Fig. S8A). This implied that transcriptional enhancement was not the primary mechanism by which chemerin-mediated CMKLR1 upregulation. Meanwhile, after inhibiting protein synthesis with cycloheximide, we found that CMKLR1 degradation rates were significantly attenuated in chemerin-overexpressing GSCs (Fig. 3A). In contrast, CMKLR1 degradation was notably accelerated in chemerin-knockdown GSCs (Fig. 3B). In addition, the increased degradation rates of CMKLR1 in chemerin-knockdown GSCs could be specifically rescued by MG132, a proteasome inhibitor. However, treatment with a lysosomal inhibitor, chloroquine (CQ), did not affect CMKLR1 degradation (Fig. 3B). The pulldown assay of exogenous CMKLR1 showed that both chemerin overexpression and rChemerin treatment led to decreased levels of CMKLR1-linked ubiquitin, whereas chemerin knockdown had the opposite effect (Fig. 3C, D; Supplementary Fig. S8B). The K48/K27 linked ubiquitin chains were found to be the main degradation mechanism of CMKLR1 in 293 cells (Supplementary Fig. S8C). This was also validated in U87MG cells (Fig. 3E). Together, these results suggest that chemerin increases CMKLR1 expression by alleviating its ubiquitin-proteasomal degradation in GBM cells.

### TNF-α is indispensable for the pro-mesenchymal effect of chemerin in GBM cells

Previous studies showed that chemerin is positively correlated with TNF-α expression [29, 30], a known potent pro-mesenchymal factor in GBM [24, 26]. Thus, we subsequently investigated whether this chemerin-mediated pro-mesenchymal effect in GBM cells was dependent on TNF-α. We detected upregulated or downregulated TNF-α protein secretion in chemerin-overexpressing or knockdown GSCs, respectively (Supplementary Fig. S9A, B). Moreover, TNF-α neutralization effectively abrogated the upregulation of mesenchymal marker expression in chemerin-overexpressing GSCs, and rTNF-α treatment recovered the effects of chemerin knockdown in GSCs. This is similar to the data showing the effects of chemerin neutralization or rChemerin treatment in the same context (Supplementary Fig. S9C, D). Together, these results suggest that TNF-α is required to mediate the pro-mesenchymal effects of chemerin in GSCs.

### TAM infiltration and mesenchymal phenotype-promoting ability is enhanced by GBM-derived chemerin

**Fig. 4:**
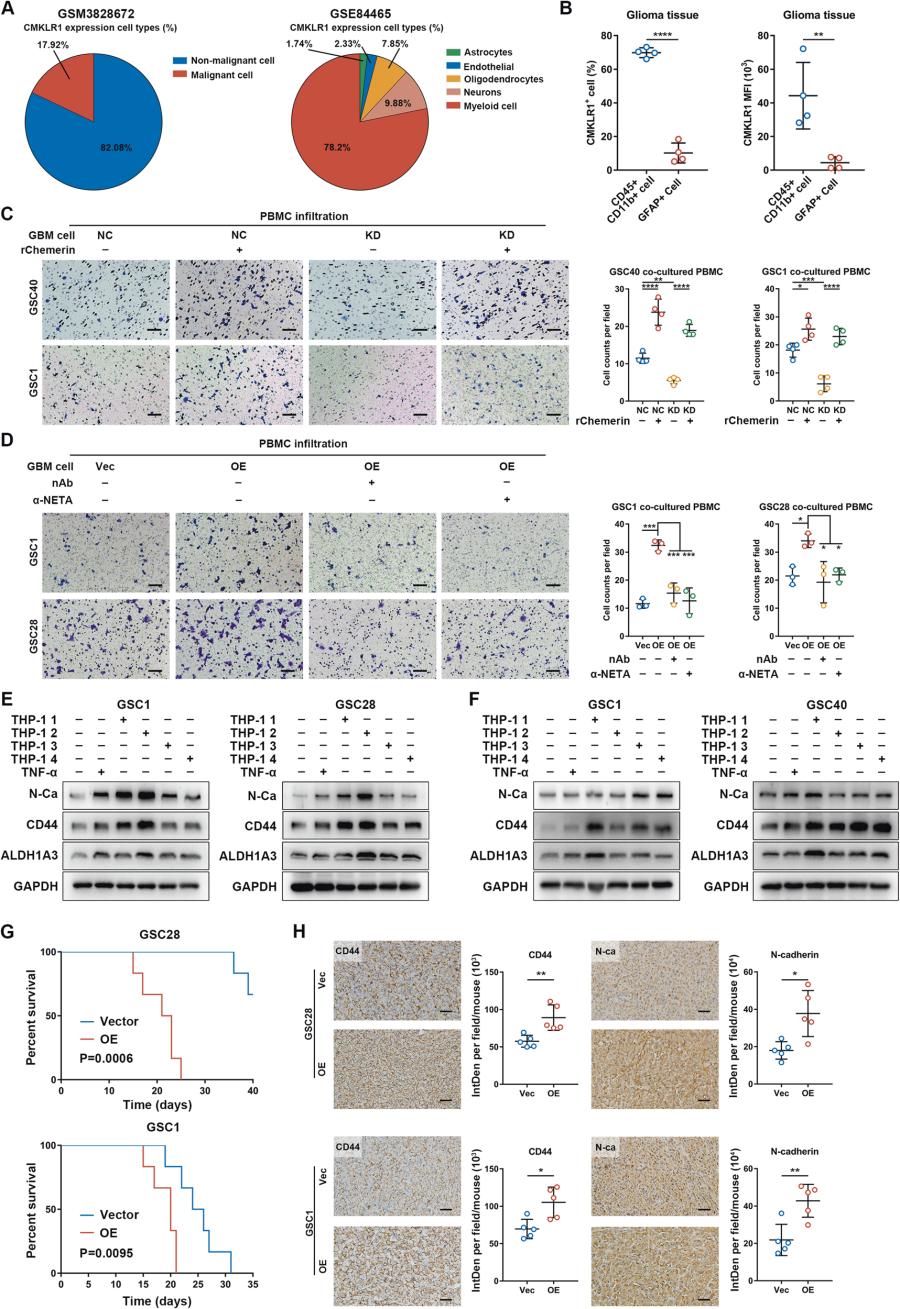
Chemerin promotes TAM infiltration and enhances their mesenchymal-promoting ability. **A** Proportion of CMKLR1-expressing cell types in GSM3828672 single-cell sequencing dataset. **B** Flow cytometry analysis of the proportion and MFI (mean fluorescence intensity) of CMKLR1 positive cells in GBM resected tissues. *n* = 4. **C** In vitro migration assay of PBMC co-cultured with indicated chemerin knockdown GSCs, treated with rChemerin. *n* = 4. **D** In vitro migration assay of PBMC co-cultured with chemerin overexpressed GSCs, treated with chemerin (neutralizing antibody) nAb or α-NETA. *n* = 3. **E** Western blotting analysis of mesenchymal markers expression in GSCs co-cultured with indicated TAMs (GSCs educated THP-1). THP-1 1: Vector GSC educated THP-1. THP-1 2: Chemerin overexpressed GSC educated THP-1. THP-1 3: Chemerin overexpressed GSC + chemerin nAb treated THP-1. THP-1 4: Chemerin overexpressed GSC + α-NETA treated THP-1. **F** Western blotting analysis of mesenchymal markers expression in GSCs co-cultured with indicated TAMs (GSCs educated THP-1). THP-1 1: Vector GSC educated THP-1. THP-1 2: Chemerin knockdown GSC educated THP-1. THP-1 3: Chemerin knockdown GSC + rChemerin treated THP-1. THP-1 4: Chemerin knockdown GSC + rTNF-α treated THP-1. *n* = 3. **G** Survival curves of mice orthotopically transplanted indicated GSCs. *n* = 6. **H** Representative IHC images and staining quantification of indicated mesenchymal markers in orthotopic GBM tissues from mice models in G. Scale bars: 50 μm. *n* = 5. Data are presented as means ± SD. Survival differences were analyzed with log-rank tests. Statistical significance in (**B**) and (**H**) was analyzed with Student’s *t* test. Statistical significance in (**C**) and (**D**) was analyzed with one-way ANOVA analyses. *p < 0.05, **p < 0.01, ***p < 0.001, ****p < 0.0001.

We then profiled the cellular expression patterns of CMKLR1 to explore cell types other than GBM cells that react to chemerin stimulation. Although individual GBM cells expressed considerable levels of CMKLR1, single cell RNA-seq data of GBM showed that non-malignant cells had higher overall CMKLR1 expression (Supplementary Fig. S10A, B) [19]. These data suggest that in addition to that in GBM cells, the chemerin/CMKLR1 axis might have expansive roles in non-malignant cells. Among the non-malignant cells, myeloid cells comprised the dominant cell type expressing CMKLR1 (Fig. 4A; Supplementary Fig. S10A) [31]. Flow cytometric analysis of freshly resected GBM samples further verified that CMKLR1 was more highly expressed in CD45^+^ CD11b^+^ TAMs than in GFAP^+^ malignant cells (Fig. 4B), suggesting the possible involvement of TAMs in the chemerin-mediated GBM-promoting mechanism.

Next, we investigated the role of chemerin in GBM–TAM interactions. GSEA based on immune cell signatures showed that only TAMs (both M1/M2 subtypes) were stably enriched in GBMs with high *RARRES2* expression (Supplementary Fig. S10C) [32]. The correlation between chemerin expression and TAM infiltration was further validated via IHC staining of GBM tissue samples (Supplementary Fig. S10D). To further determine the influence of GBM-derived chemerin on TAMs, we co-cultured GSCs with phorbol myristate acetate-differentiated peripheral blood mononuclear cell (PBMC) or HMC3 cells to transform them into TAMs. We found that chemerin knockdown in GSCs significantly decreased their TAM-recruiting ability, and these effects could be rescued by adding rChemerin in the co-culturing system (Fig. 4C; Supplementary Fig. S11A). In contrast, overexpressing chemerin in GSCs significantly increased their TAM-recruiting ability, whereas blocking the chemerin/CMKLR1 axis via chemerin neutralization or α-NETA treatment effectively reduced TAM infiltration (Fig. 4D; Supplementary Fig. S11B).

Interestingly, when TAMs were induced by chemerin-overexpressing GSCs, both inflammatory (IL-1β and TNF-α) and immunosuppressive (PD-L1 and TGF-β) factors were upregulated. However, when the chemerin/CMKLR1 axis was blocked, only immunosuppressive factors showed a consistent decreasing trend (Supplementary Fig. S12A, B). Additionally, flow cytometric analysis showed a decreased tendency of M1 polarization when TAMs were co-cultured with chemerin-overexpressing GSCs. Chemerin/CMKLR1 axis blockade also effectively increased TAM’s M1 polarization (Supplementary Fig. S12C, D). These data suggest the therapeutic potential of chemerin/CMKLR1 axis blockade to suppress the M2 polarization of TAMs.

Finally, a two-step co-culture system was established to investigate the effects of tumor-derived chemerin on the mesenchymal phenotype-promoting ability of TAMs (Supplementary Fig. S13A). Results showed that TAMs activated by chemerin-overexpressing GSCs had a higher capacity to promote mesenchymal marker expression in GSCs, whereas chemerin neutralization or α-NETA treatment in the first step of co-culturing suppressed this effect (Fig. 4E; Supplementary Fig. S13B). In contrast, TAMs exposed to chemerin-knockdown GSCs had a diminished mesenchymal phenotype-promoting ability, as evidenced by the decreased expression of mesenchymal markers in GSCs. Moreover, administering rChemerin to chemerin-knockdown GSCs in the first step of co-culturing rescued the mesenchymal phenotype-promoting ability of TAMs (Fig. 4F; Supplementary Fig. S13C). Together, these findings suggest that GBM-derived chemerin plays an important role in establishing a paracrine mesenchymal phenotype-promoting interaction between GBM cells and TAMs.

### Chemerin enhances mesenchymal features and TAM infiltration to promote GBM progression in vivo

Next, we sought to determine the malignant effects of chemerin using in vivo orthotopic GBM mouse models (i.e., those implanted with GSC1, GSC28, and GSC40 cells). Results showed that chemerin overexpression significantly increased the tumor volume in GBM-bearing mice and reduced mouse survival (Fig. 4G; Supplementary Fig. S14A, B). In contrast, chemerin knockdown significantly decreased the tumor volume in GBM-bearing mice and prolonged overall survival (Supplementary Fig. S14C, D). As expected, the expression of mesenchymal markers was increased in chemerin-overexpressing tumors but decreased in chemerin-knockdown tumors (Fig. 4H; Supplementary Fig. S14E, F). Additionally, chemerin-overexpressing GBM tissues had higher TAM infiltration (Supplementary Fig. S14G), whereas chemerin-knockdown GBM tissues showed significantly decreased TAM infiltration (Supplementary Fig. S14H). Taken together, these results indicate that chemerin plays an important role in promoting mesenchymal features and TAM infiltration in GBM.

### Chemerin/CMKLR1 axis promotes the interaction between GBM cells and TAMs by activating NF-κB signaling

**Fig. 5:**
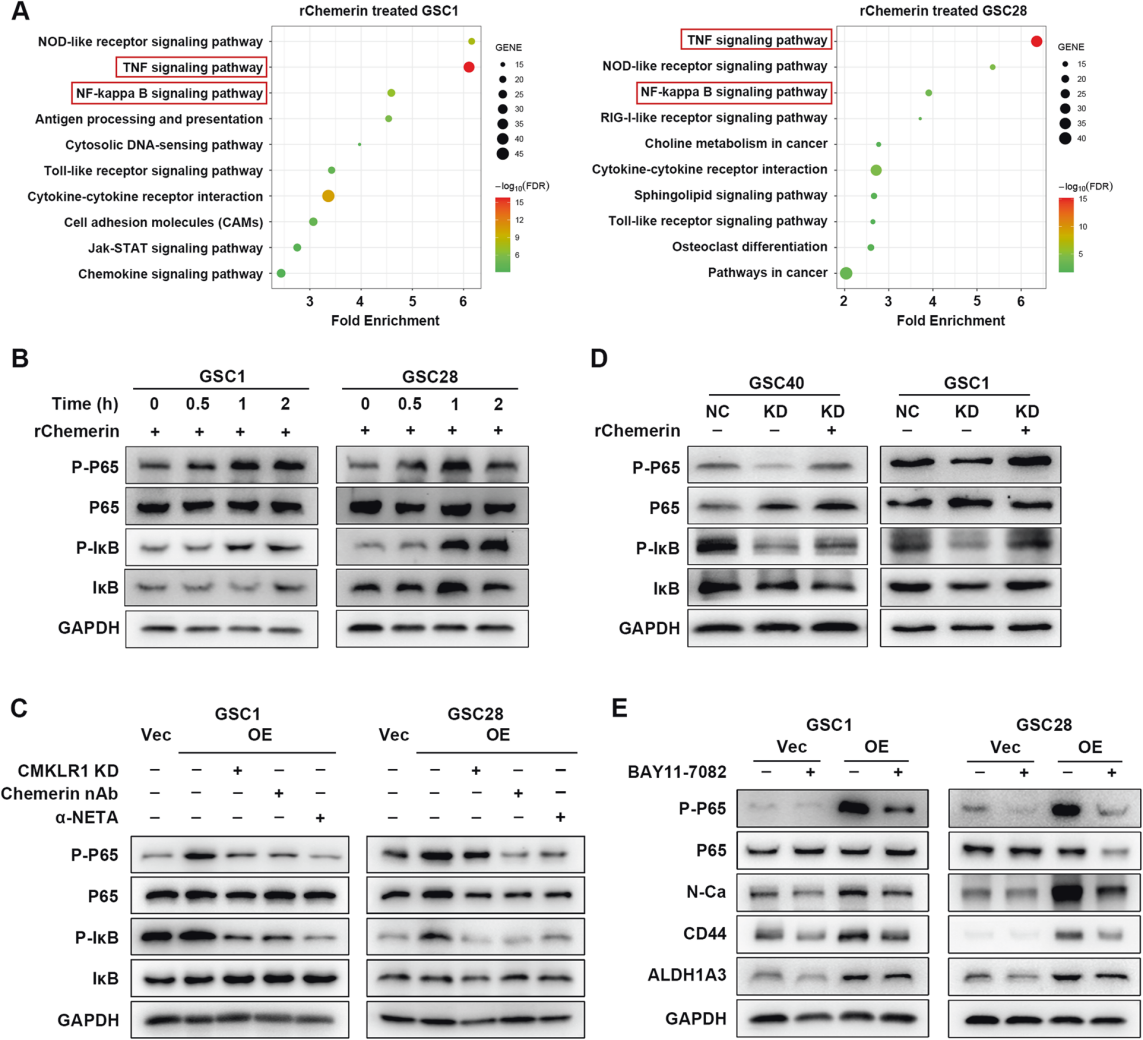
Chemerin activates NF-κB signaling to promote mesenchymal feature of GBM. **A** KEGG analysis showing top 10 pathway terms related to upregulated genes in indicated rChemerin treated GSCs. *n* = 3. **B** Western blotting analysis of time dependent NF-κB signaling changes in rChemerin treated GSCs. **C** Western blotting analysis of NF-κB signaling change in chemerin overexpressed GSCs treated with α-NETA or chemerin nAb, or with CMKLR1 knockdown. **D** Western blotting analysis of NF-κB signaling change in chemerin knockdown GSCs treated with rChemerin. **E** Western blotting analysis of NF-κB signaling and indicated mesenchymal markers expression in chemerin overexpressed GSCs treated with BAY11-7082.

To explore the mechanisms underlying the mesenchymal phenotype-promoting effects of chemerin, KEGG pathway analysis was performed with upregulated genes related to higher *RARRES2* expression, summarized based on TCGA and CGGA GBM datasets. As shown in Supplementary Fig. S9 that TNF-α was indispensable for the effect of chemerin on GBM cells, TNF signaling was significantly enriched in samples from patients with GBM with higher chemerin expression (Supplementary Fig. S15A). Expression profiles of rChemerin-treated GSCs also showed activated TNF signaling (Fig. 5A). Furthermore, we noticed a positive association between NF-κB signaling and chemerin in the profile data of patients with GBM and GSC samples (Fig. 5A; Supplementary Fig. S15A). Considering that chemerin and TNF-α have been shown to activate NF-κB signaling [33–37], which is an important pathway that regulates the mesenchymal features of glioma [24, 38], we suggest an important role for NF-κB signaling in the chemerin/CMKLR1 axis in GBM. Indeed, rChemerin treatment upregulated canonical NF-κB signaling in GSCs in a time-dependent manner (Fig. 5B). Chemerin neutralization, α-NETA treatment, and CMKLR1 knockdown effectively decreased NF-κB signaling in chemerin-overexpressing GSCs (Fig. 5C). In contrast, chemerin knockdown suppressed NF-κB signaling and rChemerin treatment effectively rescued attenuated NF-κB signaling in chemerin-knockdown GSCs (Fig. 5D). Moreover, the inhibition of NF-κB signaling remarkably decreased mesenchymal marker expression in chemerin-overexpressing GSCs (Fig. 5E), suggesting the involvement of NF-κB signaling in chemerin-mediated enhancement of mesenchymal features in GBM cells. Although previous studies showed that MAPK or PI3K-AKT signaling was the downstream effector of chemerin stimulation [39, 40], we did not observe consistent changes in these two signaling pathways in GSCs (Supplementary Fig. S15B). Additionally, the AKT signaling-specific inhibitor MK2206 could not completely abrogate the chemerin-mediated enhancement of mesenchymal features in GSCs (Supplementary Fig. S15C). These results indicate that chemerin enhances mesenchymal features the GBM cells through canonical NF-κB signaling.

Next, to explore the effect of chemerin on TAM signaling, we co-cultured THP-1-derived macrophages or HMC3 cells with either chemerin-knockdown or chemerin-overexpressing GSCs. We found that chemerin-knockdown GSCs significantly reduced NF-κB activation in TAMs co-cultured with GSCs, and rChemerin supplementation effectively rescued this effect (Supplementary Fig. S16A, B). In contrast, chemerin-overexpressing GSCs induced enhanced NF-κB signaling in TAMs, whereas chemerin neutralization and α-NETA treatment induced the opposite effect (Supplementary Fig. S16C, D). These results suggest that NF-κB, but not PI3K-AKT or MAPK, signaling is the downstream effector of GBM-derived chemerin in TAMs. Together, these results indicate the crucial role of NF-κB signaling in chemerin-mediated interactions between GBM cells and TAMs.

### Chemerin/CMKLR1 axis blockade suppresses the mesenchymal phenotype-promoting network and improves the anti-tumor TME in chemerin-overexpressing GBMs

**Fig. 6:**
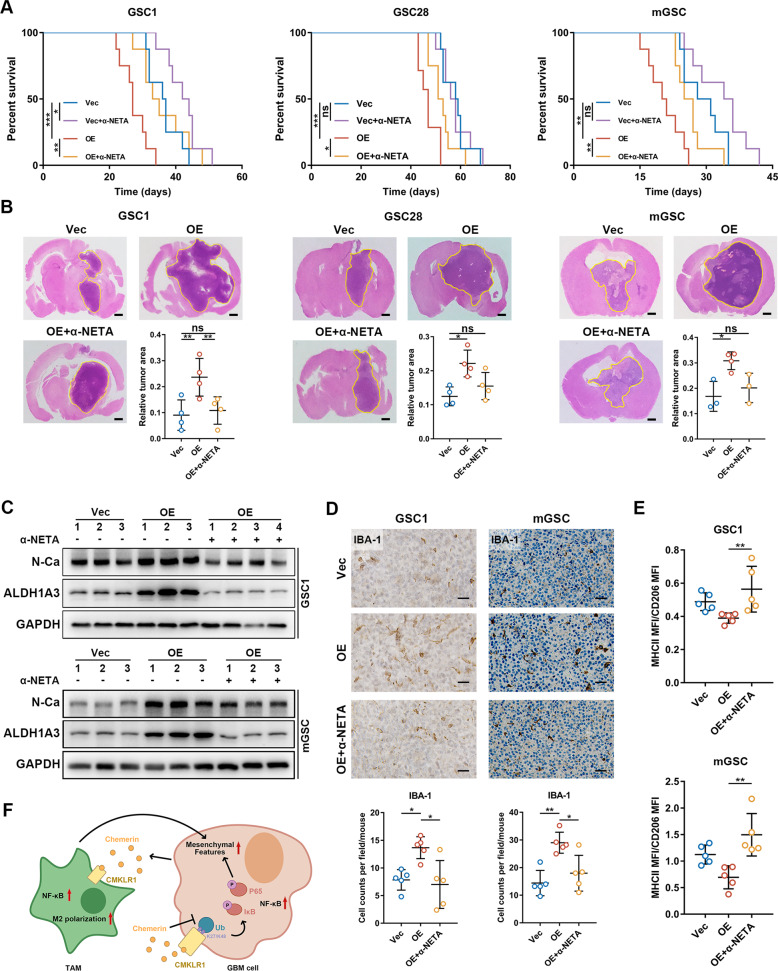
Blockade of chemerin/CMKLR1 axis significantly impairs chemerin-mediated tumor growth. **A** Survival curves of indicated orthotopic chemerin overexpressed GBM models treated with α-NETA. *n* = 8 except for GSC28 OE group, *n* = 7 in GSC28 OE group. **B** Representative H&E staining images and area quantification of the maximum mouse brain cross-section of indicated chemerin overexpressed orthotopic GBM tumors. Scale bars: 500 μm. *n* = 4 except for mGSC Vec and mGSC OE + α-NETA group, *n* = 3 in mGSC Vec and mGSC OE + α-NETA group. **C** Representative IHC staining images and quantification of IBA-1 in indicated orthotopic GBM tumor tissues. Scale bars: 50 μm. *n* = 5. **D** Flow cytometry analysis of the ratio of MHCII MFI/CD206 MFI of TAM in GSCs tumors. *n* = 5. **E** Western blotting analysis of mesenchymal markers in indicated chemerin overexpressed orthotopic GBM tumors treated with α-NETA. **F** Summary of the mechanisms of chemerin/CMKLR1 axis in GBM. Data are presented as means ± SD. Survival differences were analyzed with log-rank tests. Statistical significance in (**D**) and (**E**) was analyzed with one-way ANOVA analyses. ns not significant, *p < 0.05, **p < 0.01, ***p < 0.001.

Next, we evaluated the effect of CMKLR1 inhibition on GBM progression in three orthotopic GBM mouse models with GSC1, GSC28, and mGSCs. Chemerin-overexpressing mGSCs were constructed as shown in Supplementary Fig. S17. α-NETA treatment significantly prolonged survival and inhibited tumor growth in both immune-deficient and immune-intact orthotopic GBM models (Fig. 6A, B). Interestingly, although α-NETA treatment had considerable suppressive effects on vector-expressing GSC1 cells, it had negligible effects on vector-expressing GSC28 cells or mGSCs (Fig. 6A). This heterogeneity, in terms of the effects of α-NETA, might be explained by the mesenchymal state of GSCs and their chemerin expression level, since GSC28 is a non-mesenchymal GBM cell line and GSC28 cells and mGSCs both have negligible chemerin expression (Supplementary Fig. S2G; Supplementary Fig. S17). This highlights the specific effect of chemerin/CMKLR1 axis blockade on chemerin-highly expressing mesenchymal GBM.

We further found that the anti-tumor effect of CMKLR1 blockade was not mainly derived from influences on GBM cell proliferation or apoptosis (Supplementary Fig. S18A, B). Instead, α-NETA treatment decreased the mesenchymal features of chemerin-overexpressing GBM models and reduced their NF-kB signaling (Fig. 6C; Supplementary Fig. S19A, B). It also markedly attenuated the infiltration of total TAMs and CCR2^+^ monocyte-derived macrophages (Fig. 6D; Supplementary Fig. S19C), as well as the M2 polarization of TAMs (Fig. 6E). Moreover, chemerin- or α-NETA-stimulated mouse bone marrow derived macrophage-derived TAMs showed similar changes in infiltration capability and inflammation-related marker expression to those of human macrophage/microglia-derived TAMs (Supplementary Fig. S20A, B). This suggests the applicability and reliability of our GBM models in investigating chemerin-mediated GBM–TAM interactions regardless of cross-talk between species.

Next, as the suppression of anti-tumor adaptive immunity is a hall mark of GBM malignancy related to TAMs [41, 42], we investigated T cell population changes upon α-NETA treatment in GBM. In mGSC tumors, although α-NETA treatment did not significantly influence the T cell population component ratio, it suppressed the upregulation of PD-1 expression in CD4^+^/CD8^+^ T cells in chemerin-overexpressing tissue (Supplementary Fig. S21A, B). Simultaneously, α-NETA treatment upregulated the significantly suppressed anti-tumor functions of T cells (Supplementary Fig. S21C, D). Moreover, we detected negligible changes in peripheral immune cell populations after α-NETA treatment, which indicates the target of α-NETA in GBM tissue (Supplementary Fig. S21E, F). Taken together, these results suggest that targeting the chemerin/CMKLR1 axis is an efficient strategy to decrease mesenchymal features and boost the anti-tumor immune environment in mesenchymal GBM.

## Discussion

In this study, we suggested that abundant TAM infiltration was the primary microenvironmental feature of mesenchymal GBM; we found that chemerin was responsible for establishing a mesenchymal phenotype-promoting network between GBM cells and TAMs in a CMKLR1-dependent manner (Fig. 6F). Our findings suggest that disrupting the chemerin/CMKLR1 axis in GBM may be a potential therapeutic strategy for alleviating the mesenchymal features and suppressing GBM progression.

Chemerin could be either a pro-tumor or an anti-tumor factor in different types of cancers. Similar with our findings in GBM, the expression level of chemerin in tumor tissue or serum is negatively correlated with patient survival in cancers such as breast cancer [43, 44], ovarian cancer [45], and non-small cell lung cancer [46]. In these cancers, chemerin could facilitate their progression mainly by promoting their invasive ability [47–49], although its influence on tumor proliferation is relatively negligible [13, 50] or even suppressive [51]. This characteristic functional change is similar to what in cancers that have undergone epithelial-to-mesenchymal transformation (EMT). Through EMT, cancer cells achieve potent invasive ability but with reduced proliferative capacity [52]. To our knowledge, the present study is the first evidence connecting the motility-promoting effect of chemerin with enhancement of the mesenchymal phenotype in GBM. So far, several mechanisms have been found to facilitate phenotypic transitions in GBM, and especially enhancement of the mesenchymal phenotype, such as the TGF-β family [53], TNF-α [24], a hypoxic environment [54], and exogenous therapies [55]. Our findings suggest that chemerin comprises one of the potentially relevant mechanisms through which GBM undergoes mesenchymal transition to enhance its phenotypic heterogeneity. Along with other roles of chemerin revealed in cancer, such as angiogenesis [56] and PD-L1/PD-1 axis regulation [39, 57], focusing on chemerin/CMKLR1 axis blockade may help develop effective therapeutics targeting mesenchymal GBM.

Chemerin has three reported receptors, and the physiological effects and related signal transducing mechanisms differ significantly among them. As shown in Supplementary Fig. S6B, the effects of knocking down GPR1 or CCRL2 contrasted with those observed with CMKLR1 knockdown. This indicates that GPR1 and CCRL2 may participate in other chemerin-mediated pathological processes in GBM. Previous studies suggest that the chemerin/CMKLR1 axis is the main signal transducing pathway of chemerin underlying its effect on cancer progression [39, 57, 58]. However, the functional response induced by the chemerin/GPR1 axis is limited to arrestin recruitment and RhoA/ROCK-mediated signaling [59]. Although most known functions of GPR1 are mediated by CMKLR1 [22, 60], the biological role of GPR1 in cancers has not been well explored, despite its involvement in metabolic processes [61–64]. For CCRL2, no detectable signaling events have been observed upon chemerin stimulation [65]. However, CCRL2 may function to increase the concentration of chemerin for its efficient presentation to CMKLR1 on nearby cells, and it was found to have unique regulatory roles in neoangiogenesis [66], immune cell recruitment [67, 68], and inflammatory responses [69]. Therefore, different mechanisms and biological processes may occur depending on the receptors stimulated by chemerin. However, owing to the poor characterization in this field, more work is needed to clarify the roles of GPR1 and CCRL2 in GBM.

Interestingly, we found that chemerin overexpression in GSCs could not offset the growth difference between that observed in these cells and that in tumors transduced with the control vector in the contralateral hemisphere of mice, as shown in Supplementary Fig. S14B. This finding suggests that merely enhancing chemerin stimulation in GBM cells is not sufficient to induce a more malignant phenotype in vivo. Instead, the gradient-dependent TAM-recruitment property of chemerin allows for chemerin-overexpressing tumor sites with higher chemerin concentrations and more TAM infiltration. Considering the suggested role of TAMs in promoting mesenchymal features, we speculate that TAMs are indispensable for the effect of chemerin when inducing GBM malignancy in a paracrine manner. Blockade of the chemerin/CMKLR1 axis could be a novel therapeutic strategy to target TAMs in GBM [70].

It should be noted that the enhanced pro-mesenchymal capacity of TAMs may be derived from chemerin-induced M2 polarization; this has been well described in previous studies [39, 53, 71, 72]. However, the partitioning of M1/M2 macrophage polarization is ambiguous in the actual tumor environment [73]. The co-expression of both representative M1 and M2 genes in individual TAM is commonly observed in GBM [74]. This may help explain why both M1- and M2-subtype TAMs were significantly enriched in GBM highly expressing chemerin, according to GBM’s RNA-seq data. Moreover, the analysis of M1/M2 markers in TAMs co-cultured with chemerin-overexpressing GSCs revealed the upregulation of both M1 and M2 markers in these cells. Interestingly, we found that NF-κB signaling in TAMs was positively regulated by GBM cell-derived chemerin. In contrast to its well-known role in promoting anti-tumoral inflammatory macrophage polarization [75], NF-κB signaling in TAMs is also essential for maintaining their immunosuppressive phenotype to aid in GBM cell immune evasion [76, 77]. The ablation of NF-κB signaling results in increased M1-polarized TAMs and prolonged survival of GBM-bearing mice [78]. Considering the autocrine effect of chemerin on activating NF-κB signaling in TAMs and GBM cells, targeting the chemerin/CMKLR1 axis in GBM could be expected to exert a similar tumor suppressive effect as that with NF-κB inhibition [79].

α-NETA is described as a small molecule antagonist that blocks the interaction between β-arrestin2 and CMKLR1 to suppress CMKLR1 activation upon chemerin stimulation [28]. Although its ability to penetrate the blood–brain barrier has not been characterized, α-NETA does effectively reduce CMKLR1^+^ leukocyte infiltration into the CNS and relieves CNS autoimmune inflammatory symptoms [28]. Since that previous study, α-NETA has been employed to block the chemerin/CMKLR1 axis in CNS-related diseases, including preeclampsia [80] and neuroblastoma [81]. Moreover, α-NETA was reported to be well tolerated in mice [82]. According to our data, it barely affected the peripheral immune environment in our mouse preclinical GBM models, and α-NETA could precisely target both GBM cells and TAMs owing to the expression pattern of CMKLR1 in GBM tissue. These results indicate that the administration of α-NETA might have few side effects. Therefore, it would be worthwhile to conduct preclinical trials to further clarify the inhibitory effect of α-NETA on GBM and its potential for combination with temozolomide [83].

In conclusion, our study describes a chemerin-mediated mesenchymal phenotype-promoting network characterized by autocrine and paracrine mechanisms. The inhibition of CMKLR1 was further found to abrogate the pro-mesenchymal effects of chemerin in GBM. This highlights the potential for the chemerin/CMKLR1 axis to serve as a promising therapeutic target for GBM.

## Materials and methods

### Ethics statement

The experimental protocol was approved by the ethics committee of The First Hospital of China Medical University. Animal experiments were conducted in accordance with the China Medical University Animal Care and Use Committee guidelines and approved by the Institutional Review Board of The First Hospital of China Medical University.

### Chinese Glioma Genome Atlas (CGGA) data

The CGGA RNA sequencing (RNA-seq) cohort consisted of 325 cases for which clinical information and expression profiles were obtained from the in-house CGGA datasets (Supplementary Table S1).

### Clinical samples

For the samples obtained from The First Hospital of China Medical University, the tissues were collected immediately after tumor resection. Tissue quality for molecular testing and diagnostic accuracy was assessed by at least two experienced neuropathologists [84]. Detailed methods and RNA-seq data processing are described in the Supplementary Information.

### Cell lines and lentiviral transduction

Patient-derived primary glioma cells (GSC28, GSC1, and GSC40) were derived from freshly resected glioma tissues as previously described [85]. Sleeping Beauty (SB) mouse glioma sphere cells (mGSCs) were harvested from SB spontaneous GBM models as previously described [86]. Commercial cell lines were purchased as described in the Supplementary Information. Recombinant shRNA lentiviral particles were constructed targeting human *RARRES2* for chemerin knockdown (Sangon Biotech, Shanghai, China). *RARRES2* cDNA was cloned into a lentivirus-based vector for chemerin overexpression (Sangon Biotech). Detailed information on cell harvesting, culture, and lentiviral transfection methods are described in the Supplementary Information.

### Biological phenotype analyses

Cell proliferation assays, apoptosis analysis, co-culture system construction, and cell invasion/migration assays were employed to evaluate the biological phenotypes of TAMs and GBM cells in vitro. The detailed procedures for these experiments are provided in the Supplementary Information.

### Index detection and quantification

Immunohistochemical (IHC) staining, immunoprecipitation, quantitative real-time polymerase chain reaction, western blotting, flow cytometric analysis, and enzyme-linked immunosorbent assays were performed to quantify the indicated biological indices in this study. Detailed procedures are described in the Supplementary Information.

### Tumor xenografts

Male BALB/c nude mice and C57BL/6 N mice (6–8 weeks of age) were purchased from Charles River (Beijing, China). Detailed methods for establishing the orthotopic transplantation tumor models are described in the Supplementary Information.

### Statistical analysis

Clinical characteristics and mRNA expression profiles of The Cancer Genome Atlas (TCGA), Gravendeel (GSE16011), and Rembrandt datasets were downloaded from Gliovis (http://gliovis.bioinfo.cnio.es/) as previously described (Supplementary Table S1) [87]. All results shown are representative of at least three independent experiments. Data are expressed as means ± SDs. Software used for statistical analyses is described in the Supplementary Information. Student’s *t* tests, one-way analyses of variance, and chi-squared tests were used to assess statistical significance. All statistical tests were two-tailed. Differences in survival were analyzed using log-rank tests and Kaplan–Meier analyses. Statistical differences were considered significant at *P* < 0.05.

## Supplementary information

Supplementary information

Supplementary Figure S1

Supplementary Figure S2

Supplementary Figure S3

Supplementary Figure S4

Supplementary Figure S5

Supplementary Figure S6

Supplementary Figure S7

Supplementary Figure S8

Supplementary Figure S9

Supplementary Figure S10

Supplementary Figure S11

Supplementary Figure S12

Supplementary Figure S13

Supplementary Figure S14

Supplementary Figure S15

Supplementary Figure S16

Supplementary Figure S17

Supplementary Figure S18

Supplementary Figure S19

Supplementary Figure S20

Supplementary Figure S21

Supplementary Table S1

Supplementary Table S2

Supplementary Table S3

Supplementary Table S4

Supplementary Table S5

Supplementary Table S6

Supplementary Table S7
