# Supplementary material for: Chemerin enhances mesenchymal features of glioblastoma by establishing autocrine and paracrine networks in a CMKLR1-dependent manner
Source: Oncogene. 2022 Apr 22;41(21):3024–36. doi: 10.1038/s41388-022-02295-w (PMC9122825; doi:10.1038/s41388-022-02295-w)
Supplement: Supplementary file 1 — Supplementary information [file 41388_2022_2295_MOESM1_ESM.docx]

**Supplementary Information**

**Supplementary methods**

**RNA-seq and quality control process**

The CGGA glioma expression profile libraries were sequenced on the Illumina HiSeq 2000 platform by the 101-bp pair-end sequencing strategy. To convert original image data into sequence data, Base calling (Illumina pipeline CASAVA v1.8.2) was used. The improper reads were filtered according to standard quality control criteria below:

(1) The reads that aligned to adaptors or primers with no more than two mismatches.

(2) The reads with more than 10% unknown bases (N bases).

(3) The reads with more than 50% of low-quality bases (quality value ≤ 5) in one read.

**Read mapping and expression analysis of RefSeq genes**

Hg19 RefSeq (RNA sequences, GRCh37) was downloaded from the UCSC Genome Browser (http://genome.ucsc.edu). The reads per kilobase transcriptome per million reads (RPKM) method which could filter out the disturb of different gene lengths and sequencing discrepancies from the expression calculation was used to estimate each gene expression [1]. Then, the calculated gene expression data were utilized in gene algorithm analysis.

**Gene filter procedure**

CGGA, TCGA and Gravendeel GBM (GSE16011) cohorts were analyzed for gene screening. Firstly, 73 overlapping genes in these three datasets were sorted out, which were positively correlated with TAM score (P < 0.05, r > 0.25, xCell method https://xcell.ucsf.edu) and upregulated in mesenchymal subtype GBM in comparison with proneural subtype GBM (Adjust P < 0.05, LogFC > 1). Secondly, 52 genes putatively encoding secretory proteins in 73 overlapping genes were selected for further analysis. Then, Kaplan-Meier analysis was performed to identify that 3 out of the 52 genes had prognostic significance (P < 0.01) in all CGGA, TCGA and Gravendeel GBM cohorts. After that, Protein-protein interaction analysis (www.string-db.org) and Co-expression analysis (www.coexpedia.org) were performed to identify prognostic genes working independently. Finally, *RARRES2*, which encoded chemerin, was identified as a key mediator to facilitate the crosstalk between mesenchymal phenotype and TAMs in GBM (Supplementary Table S3).

**Cell lines and cell culture**

Primary glioma sphere cells GSC1, GSC28, and GSC40 were derived from fresh glioma tissues resected from three patients at the First Hospital of China Medical University as previously described [2, 3]. Human GSCs were cultured in stem cell medium (Neurobasal-A medium with B27 supplement, 10 ng/ml EGF and 10 ng/ml FGF) at 37 °C in a humidified 5% CO_2_. mGSC was derived from sleeping beauty transposon (SB) spontaneous GBM model as previously described [4]. Briefly, PEI/DNA complexes (vivo-jetPEI=0.08ul, total of DNA=0.5μg, component ratio of DNA=1:2:2:2 (PT2/C-Luc//PGK-SB13: pT/CMV-SV40-LgT: pT/Caggs-NRASV12: pT/Caggs-RARRES2-MYC) per mouse) were prepared according to nucleic acid delivery protocol to achieve N/P ratio of 8 (Polyplus Transfection). PT2/C-Luc//PGK-SB13 (Addgene plasmid # 20207), pT/CMV-SV40-LgT (Addgene plasmid # 20204), and pT/Caggs-NRASV12 (Addgene plasmid # 20205) were gifts from John Ohlfest. Chemerin overexpression plasmid, pT/Caggs-Rarres2-MYC, was constructed as follows: Gene fragment of Rarres2-Myc-DDK-tagged (NM_027852: ATGAAGTTCTTGCTGATCTCCCTAGCCCTATGGCTGGGCACAGTGGGCACACGTGGGACAGAGCCCGAACTCAGCGAGACCCAGCGCAGGAGCCTACAGGTGGCTCTGGAGGAGTTCCACAAACACCCACCTGTGCAGTTGGCCTTCCAAGAGATCGGTGTGGACAGAGCTGAAGAAGTGCTCTTCTCAGCTGGCACCTTTGTGAGGTTGGAATTTAAGCTCCAGCAGACCAACTGCCCCAAGAAGGACTGGAAAAAGCCGGAGTGCACAATCAAACCAAACGGGAGAAGGCGGAAATGCCTGGCCTGCATTAAAATGGACCCCAAGGGTAAAATTCTAGGCCGGATAGTCCACTGCCCAATTCTGAAGCAAGGGCCTCAGGATCCTCAGGAGTTGCAATGCATTAAGATAGCACAGGCTGGCGAAGACCCCCACGGCTACTTCCTACCTGGACAGTTTGCCTTCTCCAGGGCCCTGAGAACCAAAACGCGTACGCGGCCGCTCGAGCAGAAACTCATCTCAGAAGAGGATCTGGCAGCAAATGATATCCTGGATTACAAGGATGACGACGATAAG) was constructed and cloned to plasmid pT/Caggs-NRASV12 by 5' KasI and 3' NsiI (using recombination method) to replace Nras gene expression domain in the original plasmid. Two microliters of PEI/DNA complexes (0.5 μg/μl) were administered at +1.5AP, +0.7ML, and −1.5DV from lambda of neonatal mouse which was placed on ice for three minutes for anesthesia before being secured in a stereotaxic frame (Stoelting). One month later, mGSCs were isolated from spontaneous GBM tissue and cultured in stem cell medium (Neurobasal-A medium with B27 supplement, 10 ng/ml EGF and 10 ng/ml FGF) at 37 °C in 5% CO_2_.

THP-1 (National Collection of Authenticated Cell Cultures, China, TCHu 57) was maintained in RPMI 1640 medium (RPMI). HMC3 (Procell Life Science&Technology Co, China, CL-0620) was maintained in MEM (Gibco, 11095080). 293 (National Collection of Authenticated Cell Cultures, GNHu18), U87MG (National Collection of Authenticated Cell Cultures, TCHu138), U251MG (National Collection of Authenticated Cell Cultures, TCHu58), and NHA (CMBIO, China) was maintained in DMEM. All the above culture medium was supplemented with 10% heat-inactivated fetal bovine serum (FBS) (Gibco) and 1:1000 Penicillin-Streptomycin (Gibco) in a humidified 5% CO_2_ atmosphere at 37∘C. Mycoplasma tests of the cells were performed monthly using MycoAlert Mycoplasma Detection Kit (Lonza, Switzerland). Cells used for the experiments were limited to less than 12 passages. Supernatant was collected from cells cultured in FBS-free culturing medium for 24h with or without additional treatment. 400 ng/ml mouse chemerin antibody (AF2325, R&D systems, USA), which has approximately 20% cross-reactivity with recombinant human chemerin according to the instruction, was used to neutralize GBM cell-derived chemerin. 0.04 μg/ml human TNF-alpha antibody (MAB610, R&D systems) was used to neutralize GBM cell-derived TNF-alpha. 7μg/ml α-NETA (HY-138097, MCE) was used for CMKLR1 inhibition.

**RNA isolation and sequencing**

Total RNA from GSCs was isolated by Trizol and sent to Novogene Company (Beijing, China) for RNA sequencing as previously described [5]. After obtaining the fragments per kilobase of exon model per million mapped fragments (FPKM) from raw RNA-Seq data, the differently expressed genes (DEGs) analysis was performed using the 'DEseq2' R package (|log 2 FC|>0.5 and FDR < 0.05), and KEGG analysis was performed.

**Constructing chemerin knockdown or overexpression GSCs**

GSCs were infected less than 24h in complete culture solution containing 10 μg/ml polybrene with lentiviral carrying chemerin knockdown shRNA or overexpression vector designed and constructed by Sangon Biotech, China. Briefly, lentivirus for knocking down chemerin in GSCs was constructed by loading easy-siRNA targeting RARRES2: CTGGGCTCTGAGGACAAAGTT to GV112 vector via AgeI/EcoRI enzyme digestion. Random easy-siRNA sequence: TTCTCCGAACGTGTCACGT was constructed as negative control. Lentivirus for overexpressing Chemerin in GSCs was constructed by loading amplified RARRES2 gene fragment to GV260 vector via BamHI/AgeI enzyme digestion. Primers for amplifying RARRES2 gene fragment were listed in Supplementary Table S6. The transfected cells were then selected using 3μg/ml puromycin for 15 days. The efficiency of chemerin knockdown or overexpression was assessed by RT-qPCR. GSCs in which chemerin overexpressed more than 2 folds or reduced by over 70% compared with control cells were achieved for further experiments.

**Gene knockdown in GSCs**

siRNAs targeting human CMKLR1, GPR1, and CCRL2 were designed and constructed by Sangon Biotech, China (Supplementary Table S6). Lipofectamine™ 3000 Reagent (Invitrogen, USA) was used to transfect siRNAs into GSCs following manufacture’s instruction. The transfection efficiency was confirmed by RT-qPCR at 48h or western blotting analysis at 72h.

**BMDM preparation**

Bone marrow-derived macrophages (BMDM) were generated from 8-12 weeks old C57BL/6 mice. Briefly, total bone marrow cells were flushed out from femur and tibia using injection syringe. Then, cells were washed with PBS twice and erythrocyte was lysed. The remaining cells were resuspended at the concentration of 5×10^6^ cells/ml in RPMI 1640 medium supplemented with 10% FBS and 1% penicillin/streptomycin in 5% CO_2_ at 37°C. Mouse macrophage colony-stimulating factor (R&D system, 416-ML) was added to the culturing medium at 20 ng/ml and cells were incubated for 7 days. Non-adherent cells were discarded, and adherent BMDM was harvested for the following experiments.

**PBMC preparation**

Human peripheral blood mononuclear cells (PBMC) were isolated from venous blood of donors with brain diseases collected in EDTA tubes (BD Vacutainer), using Ficoll density gradient centrifugal separation method (GE Healthcare, 17-1440-02). PBMC were then incubated overnight in RPMI-1640 supplemented with 10% FBS and 1% penicillin/streptomycin. Then PBMC was allowed to be differentiated into resting macrophages by culturing cells in culturing medium containing 50 ng/ml phorbol-12-myristate-13-acetate (PMA; Sigma) for 72h, and adherent cells were harvested for the next experiments.

**Constructing GBM-TAM co-culturing system**

TAMs were obtained by co-culturing 1×10^5^ THP-1, HMC3, PBMC, or BMDM per well (6-well transwell inserts) with chemerin overexpressed or knockdown GSCs pre-seeded in nether receiver wells (6 well plate) for 48h (1^st^ co-culturing). Then, to investigate mesenchymal phenotype-promoting ability of TAMs, 6-well transwell inserts with educated TAMs were transferred to another 6 well plate which was pre-seeded with 5×10^5^ normal GSCs and co-cultured for another 48h (2^nd^ co-culturing). The invasive and migratory abilities and mesenchymal marker expression of GSCs from the 2^nd^ co-culturing were evaluated.

**Cell invasion and migration assays**

After dissociated into single cells solution, chemerin overexpressed or knockdown GSCs or TAMs were resuspended in 200 ul serum free culture medium and seeded into 24-well transwell inserts (5.0 μm) at a concentration of 5×10^4^ cells per well. The inserts were then incubated at 37^o^C and the cells were allowed to invade or migrate through the Matrigel-coated (invasion) or none-coated (migration) filters toward the nether receiver wells filled with 600 μl 10% FBS medium (for GSCs to evaluate their invasion or migration abilities), or with indicated conditioned medium or pre-seeded with indicated GSCs (for THP-1, PBMC, HMC3, and BMDM to evaluate their infiltration ability). After 24h’s incubation, non-migrated cells at the top of the transwell filter were removed using a cotton swab. The invaded or migrated cells were fixed and stained with 1% crystal violet. Then stained cells were photographed using a microscope (Olympus, Japan) and counted as per field of view with Image J software (National Institutes of Health, USA).

**Quantitative real-time PCR**

Total RNA was extracted with TRIzol (TaKaRa, Japan) following manufacturer’s instruction. The RNA was reverse-transcribed into cDNA using PrimeScript™ RT Master Mix (RR036A, Takara). Quantitative PCR was performed using TB Green® Premix Ex Taq™ (RR420A, Takara) in a LightCyclerR480 (Roche Diagnostics Ltd., Basel, Switzerland) under identical amplification conditions. Each reaction was performed in triplicate. The primers’ sequencing was listed in Supplementary Table S6. Target genes expression was normalized to 18S or mGapdh levels and quantified with the 2-△△Ct method.

**Enzyme-linked immunosorbent assay (ELISA)**

Chemerin and TNF-α concentration in healthy doners’ and glioma patients’ serum or in the 24h supernatant of cultured GBM cells were measured by commercial Human Chemerin ELISA Kits (DCHM00, R&D Systems) and Human TNF-alpha Quantikine ELISA Kit (DTA00D, R&D Systems), respectively. Optimal standard curves were applied to individual assays and the absorbance values were detected at 540 nm using a microplate reader.

**Ubiquitination analysis and immunoprecipitation**

Chemerin overexpressed or knockdown GBM cells were treated with 50 nmol/L chloroquine, 3-MA or 10uM MG132 for 6 hours before CHX (100ug/ml) treatment for the next indicated hours. Cells were then harvested and lysed by Cell lysis buffer for western blotting analysis to detect CMKLR1 degradation. To assess the polyubiquitin chains bound to CMKLR1, 293 or U87MG cells were transfected with HA-Ubiquitin-WT (#17608, Addgene), HA-Ubiquitin K63 (#17606, Addgene), pET3a-Ub-K63R (#18898, Addgene) which was HA-tagged by Genewiz, China, HA-Ubiquitin K48R (#17604, Addgene), HA-Ubiquitin K48 (#17605, Addgene), pRK5-HA-Ubiquitin-K29 (#17602, Addgene), pRK5-HA-Ubiquitin-K29R (#17602, Addgene), pRK5-HA-Ubiquitin-K11 (#121152, Addgene), pRK5-HA-Ubiquitin-K11R (#121154, Addgene), pRK5-HA-Ubiquitin-K27 (#22902, Addgene), pRK5-HA-Ubiquitin-K27R (#121155, Addgene), pRK5-HA-Ubiquitin-K6 (#22900, Addgene), pRK5-HA-Ubiquitin-K6R (#121153, Addgene) for 72h. CMKLR1-Flag plasmid was designed and constructed by GENECHEM, China. Briefly, CMKLR1 gene fragment was achieved by PCR amplification using primers listed in Supplementary Table S6. CMKLR1 gene fragment was loaded to CV702 vector at BamHI/HindIII enzyme cutting site. Cells were harvested and lysed for immunoprecipitating using anti-Flag antibody (14793S, CST). Then, the lysates were incubated for 2 h at room temperature with Protein A/G Magnetic Beads (#88802, Thermo Scientific™). After three times of the wash, purified proteins were subjected to immunoblotting to detect the protein levels of HA tagged Ub.

**Western blotting analysis**

The samples were shattered in Cell lysis buffer (P0013J, Beyotime) containing 1% PMSF (ST506, Beyotime) using ultrasonic cell disruptor to extract proteins. The isolated proteins were separated by electrophoresis in SDS-PAGE (P0012A, Beyotime) and transferred to PVDF membrane (FFP28, Beyotime). After being blocked with 5% skim milk for 2 hours, the membranes were incubated with primary antibodies overnight at 4 °C. The primary antibodies used in this study were as follows:

Chemerin (ab72965, Abcam), CMKLR1 (ab64881, Abcam), Akt (#4685, CST), p-Akt (#4060, CST), IKB-α (ab32518, Abcam), P-IKB-α (ab133462, Abcam), P65 (#8242, CST), P-P65 (#3033, CST), p44/42 MAPK (Erk1/2) (#4695, CST), Phospho-p44/42 MAPK (Erk1/2) (#4370, CST), N-Cadherin (#13116, CST), CD44 (#37259, CST), ALDH1A3 (ab129815, Abcam), Ubiquitin (10201-2-AP, Proteintech), HA Tag (66006-2-Ig, Proteintech), Flag Tag (CST, 14793S), and GAPDH (ab8245, Abcam). The membranes were then incubated with the appropriate HRP-conjugated secondary antibodies (SA00001-1 and SA00001-2, Proteintech) for 1h. The immunoreactive bands were visualized using Western Blotting Luminol Reagent (sc-2048, Santa Cruz Biotechnology) and quantified using the Image J software (National Institutes of Health, Bethesda, MD, USA).

**Tumor xenografts and mouse treatment**

Animal experiments were conducted in accordance with the China Medical University Animal Ethics Committee guidelines and approved by the Institutional Review Board of our hospital. For orthotopic GBM models, Chemerin overexpressed or knockdown GSCs were adjusted to 1 ×10^5^ in 3μl of PBS and implanted intracranially into anaesthetized mice as previously described [6]. After 3 days of tumor implantation, chemerin overexpressed or knockdown tumor bearing mice were randomly divided into α-NETA treatment and control groups. α-NETA was injected intraperitoneally (once every other day, 30 mg/kg) in treatment groups for two weeks and control groups were treated with DMSO. The death time of the orthotopic tumor-bearing mice were recorded for survival analysis. The whole brains of tumor-bearing mice were removed 20 days after tumor implantation for further examinations. The H&E sections with the largest tumor cross-sectional area was selected for tumor size measurement in intracranial glioma models.

**Immunohistochemistry staining**

As previously described [7], the human and mouse paraffin-embedded tumor tissues were cut into 5-um sections and mounted onto glass slides. After deparaffinization and antigen retrieval, blockade of endogenous peroxidase activity was performed by using 3% hydrogen peroxide (H_2_O_2_). The sections were then incubated with the following primary antibodies overnight at 4℃, respectively:

Chemerin (10216-1-AP, Proteintech), IBA-1(ab178846, Abcam), CCR2 (ab273050, Abcam), N-Cadherin (#13116, CST), CD44 (#37259, CST), Vimentin (#5741, CST).

After rinsed the sections with PBS, General purpose SAP kit (SAP-9100, ZSGB-BIO) and DAB color rendering kit (ZLI-9017, ZSGB-BIO) was used for antigen visualization. Stained tissues were imaged with a light microscope (Nikon Eclipse TS100)/ (Olympus Optical Co., Ltd., Tokyo, Japan). Integrated option density was quantified using the Image J software. German immunohistochemical score (GIS) was evaluated by at least two experienced pathologists [8].

**Flow cytometry**

Orthotopic GBM tissues were resected and digested to acquire mononuclear cell suspension as previously described [9]. Mononuclear cell suspension or GSCs co-cultured THP-1 or PBMC were blocked firstly with hFc Block (#564219, BD Pharmingen) or mFc Block (#553141, BD Pharmingen), and then incubated at 4℃ for 30 min with the following fluorescein-conjugated specific antibodies, respectively:

mCD45 (#563891, BD Pharmingen), hCD45 (#563204, BD Pharmingen), hGFAP (#560298, BD Pharmingen), mCD11b (#553310, BD Pharmingen), hCD11b (#340712, BD Pharmingen), F4/80 (#565411, BD Pharmingen), mMHCII (#557000, BD Pharmingen), hMHCII (#340549, BD Pharmingen), mCD206 (FAB25351R-100UG, R&D Systems), hCD206 (#564062, BD Pharmingen), CD3e (#551163, BD Pharmingen), CD4 (#552051, BD Pharmingen), CD8a (#553030, BD Pharmingen), and PD-1 (#562671, BD Pharmingen).

IFN-γ (#554412, BD Pharmingen) and TNF-α (#554420, BD Pharmingen) staining was performed using Flow Cytometry Fixation & Permeabilization Buffer Kit I (#FC009, R&D Systems). Isotype-matched non-specific immunoglobulins were used as controls. Dead cells were excluded by 7-ADD staining. After washing twice with staining buffer, cells were resuspended in 400ul of PBS and analyzed using a BD LSRFortessa flow cytometer (BD Biosciences, San Jose, CA, USA). Results were analyzed and processed with FlowJo V10 software (TreeStar, USA).

**Statistics**

All results presented are representative of at least three independent experiments. Student’s *t* tests, One-way analyses, and Chi-squared tests were used to assess the statistical significance among different groups. All statistical tests were two-tailed. Differences in survival were analyzed using log-rank tests and Kaplan–Meier analysis. Statistical analyses were performed with SPSS, version 19.0 (SPSS Inc., Chicago, IL, USA) or using GraphPad Prism. The composition of immune cells was decoded by the ssGSEA [10] and CIBERSORT [11] algorithms. The GSVA algorithm was used to estimate mesenchymal status based on six gene sets from the MSigDB database (Supplementary Table S2). ssGSEA, GSVA and CYBERSORT algorithms were calculated via the statistical software R. The scores of hypoxia-independent mesenchymal type cells (MES1), hypoxia-dependent mesenchymal type cells (MES2), oligodendrocytic precursor cells (OPC1) and astrocytes (AC) were calculated for malignant cells according to established algorithms based on specific clustered gene sets [3]. Unsupervised hierarchical clustering was performed with R (pheatmap package). For all asterisks presented in this article, they were represented as following: ns not significant, ^*^ P < 0.05, ^**^ P < 0.01, ^***^ P < 0.001, ^****^ P < 0.0001.

**Reference**

1. Mortazavi A, Williams BA, McCue K, Schaeffer L, Wold B. Mapping and quantifying mammalian transcriptomes by RNA-Seq. Nat Methods. 2008;5(7):621-8.

2. Bowman RL, Wang Q, Carro A, Verhaak RGW, Squatrito M. GlioVis data portal for visualization and analysis of brain tumor expression datasets. Neuro-oncology. 2017;19(1):139-41.

3. Neftel C, Laffy J, Filbin MG, Hara T, Shore ME, Rahme GJ, et al. An Integrative Model of Cellular States, Plasticity, and Genetics for Glioblastoma. Cell. 2019;178(4).

4. Wiesner SM, Decker SA, Larson JD, Ericson K, Forster C, Gallardo JL, et al. De novo induction of genetically engineered brain tumors in mice using plasmid DNA. Cancer Res. 2009;69(2):431-9.

5. Wang M, Zhang C, Zheng Q, Ma Z, Qi M, Di G, et al. RhoJ facilitates angiogenesis in glioblastoma via JNK/VEGFR2 mediated activation of PAK and ERK signaling pathways. Int J Biol Sci. 2022;18(3):942-55.

6. Han S, Meng L, Jiang Y, Cheng W, Tie X, Xia J, et al. Lithium enhances the antitumour effect of temozolomide against TP53 wild-type glioblastoma cells via NFAT1/FasL signalling. Br J Cancer. 2017;116(10):1302-11.

7. Han S, Xia J, Qin X, Han S, Wu A. Phosphorylated SATB1 is associated with the progression and prognosis of glioma. Cell Death Dis. 2013;4:e901.

8. Remmele W, Schicketanz KH. Immunohistochemical determination of estrogen and progesterone receptor content in human breast cancer. Computer-assisted image analysis (QIC score) vs. subjective grading (IRS). Pathol Res Pract. 1993;189(8):862-6.

9. Becher B, Waisman A. T-helper cells: methods and protocols. Preface. Methods Mol Biol. 2014;1193.

10. Jia Q, Wu W, Wang Y, Alexander PB, Sun C, Gong Z, et al. Local mutational diversity drives intratumoral immune heterogeneity in non-small cell lung cancer. Nat Commun. 2018;9(1):5361.

11. Newman AM, Liu CL, Green MR, Gentles AJ, Feng W, Xu Y, et al. Robust enumeration of cell subsets from tissue expression profiles. Nat Methods. 2015;12(5):453-7.

**Supplementary figure legends**

**Supplementary Figure S1. TAM score is positively correlated with GBM mesenchymal phenotype.**

(A) The heat map showing 28 immune cell signature expression patterns between two classified GBM subtypes (Cluster I and Cluster II) in TCGA GBM cohort. (B) Immune cell infiltration fractions (n = 22) were compared between mesenchymal and proneural GBM. (C and D) The heat map showing mesenchymal markers/proneural markers/mesenchymal related signatures in CGGA and TCGA GBM cohorts. Samples were ordered from the lowest to the highest macrophage score and divided to three clusters termed High/Medium/Low. Statistical significance in A and B was analyzed with Student’s *t* test. ^*^ P < 0.05, ^***^ P < 0.001, ^****^ P < 0.0001.

**Supplementary Figure S2. Chemerin is highly expressed in mesenchymal GBM cells.**

(A) *RARRES2* expression was compared between patients with LGG and GBM in CGGA and TCGA glioma cohorts. (B) ELISA analysis of chemerin concentration in serum of healthy donors and glioma patients. Normal sample, n = 6; LGG sample, n = 8; GBM sample, n = 7. (C) The histogram showing the fraction of high and low *RARRES2* expression groups in chr.7 amplification or chr.7 normal GBM samples in TCGA. The median value of *RARRES2* expression was used to divide patients into low or high *RARRES2* expression groups. (D) Table showing multivariate cox analysis of *RARRES2* expression and chr.7.gain/chr.10.loss CNV event in TCGA GBM. (E) *RARRES2* expression levels were compared among three subtypes of GBM. MES = mesenchymal, PN = proneural. CL = classical. (F) *RARRES2* expression was compared in malignant cell populations with high (> median value) and low (< median value) MES1, MES2, OPC1, and AC scores, respectively. (G) Table showing molecular subtype identification, chemerin expression, and artificial gene modulation types of indicated GBM cells. (H) The histogram showing the fraction of whether lipid metabolism related GSVA scores was more than that of U87MG in GSCs. Data is presented as means ± SD. Statistical significance in A and F was analyzed with Student’s *t* test. Statistical significance in B and E was analyzed with one-way ANOVA. Chi-square test was used in C. ns = not significant, ^*^ P < 0.05, ^***^ P < 0.001, ^****^ P < 0.0001.

**Supplementary Figure S3. Chemerin does not have mesenchymal promoting effects in GBM cells with high mesenchymal feature**

(A) Western blotting analysis of indicated mesenchymal markers expression in rChemerin treated GSCs. (B) Representative images and cell count quantification of the migration and invasion analysis of rChemerin treated GSC40. n = 3. (C and D) ELISA analysis of chemerin expression in the supernatant from chemerin overexpressed or knockdown GSCs. n = 3. Data is presented as means ± SD. Statistical significance in B, C, and D was analyzed with Student’s *t* test. ns = not significant, ^*^ P < 0.05, ^**^ P < 0.01.

**Supplementary Figure S4. Chemerin strengthens mesenchymal features of GBM cells.**

(A and D) Real-time PCR analysis of indicated MT-TFs in chemerin overexpressed or knockdown GSCs. n = 3. (B and C) Representative images and cell count quantification of migration and invasion analysis of chemerin knockdown GSCs, treated with rChemerin or rTNF-α. n = 3. Scale bars: 50 μm. Data is presented as means ± SD. Statistical significance in A and D was analyzed using Student’s *t* test. Statistical significance in B and C was analyzed using one-way ANOVA. ^*^ P < 0.05, ^**^ P < 0.01, ^***^ P < 0.001, ^****^ P < 0.0001.

**Supplementary Figure S5. CMKLR1 expression is important for chemerin to elicit its prognostic value in GBM.**

(A) *CMKLR1* relative expression levels were compared among three molecular subtypes in TCGA, Gravendeel, and Rembrandt GBM cohorts. (B) Kaplan–Meier survival analysis of GBM based on *CMKLR1* high (> median value) and low (< median value) expression levels. (C) Kaplan–Meier survival analyses of GBM patients divided into four groups based on the expression levels of *RARRES2* and *CMKLR1*. Data is presented as means ± SD. Differences in survival were analyzed using log-rank tests. Statistical significance in A was analyzed with one-way ANOVA. ns = not significant, ^*^ P < 0.05, ^**^ P < 0.01, ^****^ P < 0.0001.

**Supplementary Figure S6. CMKLR1 is the indispensable receptor for chemerin to elicit its mesenchymal promoting effects in GBM cell.**

(A and B) Real-time PCR analysis of the knocking down efficacy of candidate receptors of chemerin (GPR1, CCRL2, and CMKLR1) and indicated MT-TFs expression in GSC1. n = 3. (C) Western blotting analysis of indicated mesenchymal markers or CMKLR1 expression in receptors knockdown GSCs. Data is presented as means ± SD. Statistical significance in A and B was analyzed with one-way ANOVA. ns = not significant, ^*^ P < 0.05, ^**^ P < 0.01, ^***^ P < 0.001, ^****^ P < 0.0001.

**Supplementary Figure S7. CMKLR1 knockdown, chemerin neutralization, and α-NETA treatment elicit similar suppressive effect on mesenchymal features of GBM cells.**

(A) Representative images and cell count quantification of the migration and invasion analysis of α-NETA treated, chemerin nAb treated, and CMKLR1 knockdown chemerin overexpressed GSCs. n = 3. (B) Western blotting analysis of mesenchymal markers expression in chemerin overexpressed GSCs treated with α-NETA or chemerin nAb, or with CMKLR1 knockdown. Data is presented as means ± SD. Statistical significance in A was analyzed with one-way ANOVA. ^*^ P < 0.05, ^**^ P < 0.01, ^***^ P < 0.001, ^****^ P < 0.0001.

**Supplementary Figure S8. Chemerin suppresses K27/K48 ubiquitin chain mediated CMKLR1 degradation in GBM cells.**

(A) Real-time PCR and western blotting analysis of CMKLR1 expression in chemerin overexpressed or knockdown GSCs. n = 6. (B) The pulldown assay of Flag-tagged CMKLR1 in rChemerin treated GSCs. The protein levels of ubiquitin were evaluated by western blotting analysis. (C) The pulldown assay of exogenous Flag-tagged CMKLR1 in 293 cells with HA-tagged ubiquitin chains overexpression (WT, K6 only, K6R mutant, K27 only, K27R mutant, K29 only, K29R mutant, K11 only, K11R mutant, K48 only, K48R mutant, K63 only, K63R mutant). The protein levels of HA were evaluated by western blotting. Data is presented as means ± SD. Statistical significance in A was analyzed using Student’s t test. ns = not significant.

**Supplementary Figure S9. TNF-α mediates chemerin’s mesenchymal promoting effect in GBM cells.**

(A and B) ELISA analysis of TNF-α expression in the supernatant from chemerin overexpressed or knockdown GSCs. n = 3. (C) Western blotting analysis of indicated mesenchymal markers expression in chemerin or TNF-α neutralized chemerin overexpressed GSCs. (D) Western blotting analysis of indicated mesenchymal markers expression in rChemerin or rTNF-α treated chemerin knockdown GSCs. Data is presented as means ± SD. Statistical significance in A and B was analyzed with Student’s *t* test. ^**^ P < 0.01.

**Supplementary Figure S10. TAMs were preferentially infiltrated in chemerin highly expressed GBM.**

(A) *t*-distributed stochastic neighbor embedding (*t*SNE) plot of *CMKLR1* in GSM3828672 single-cell RNA sequencing GBM dataset. (B) Western blotting analysis and relative gray value quantification of CMKLR1 expression in GBM cell lines, microglia/macrophage cell lines, and TAMs. (C) GSEA analysis of immune-related cell gene signatures in GBM patient group with *RARRES2* high-expression (> median value). (D) Representative IHC images and staining quantification of IBA-1^+^ TAMs in GBM samples with low and high chemerin expression levels. Scale bars: 50 μm. Low chemerin expression group, n = 13; High chemerin expression group, n = 11. Data is presented as means ± SD. Statistical significance in C was analyzed with Student’s *t* test. ^*^ P < 0.05.

**Supplementary Figure S11. Chemerin upregulates tumor-associated microglia infiltration.**

(A) *In vitro* migration assay of MHC3 co-cultured with indicated chemerin knockdown GSCs, treated with rChemerin. n = 3. (B) *In vitro* migration assay of HMC3 co-cultured with chemerin overexpressed GSCs, treated with chemerin nAb or α-NETA. n = 3. Data is presented as means ± SD. Statistical significance in A and B was analyzed with one-way ANOVA. ^*^ P < 0.05, ^**^ P < 0.01, ^***^ P < 0.001, ^****^ P < 0.0001.

**Supplementary Figure S12. Chemerin/CMKLR1 axis blockade suppresses M2 polarization of TAMs.**

(A and B) Real-time PCR analysis of the inflammatory factors (IL-1β and TNF-α) and immunosuppressive factors (TGF-β and PD-L1) in HMC3 or PBMC co-cultured with indicated GSCs. n = 3. (C and D) Flow cytometry analysis of the ratio of MFI (mean fluorescence intensity) of MHCII and CD206 in HMC3 or PBMC co-cultured with indicated GSCs. n = 3. Data is presented as means ± SD. Statistical significance in A to D was analyzed with one-way ANOVA. ns = not significant, ^*^ P < 0.05, ^**^ P < 0.01, ^***^ P < 0.001, ^****^ P < 0.0001.

**Supplementary Figure S13. Chemerin increases mesenchymal promoting ability of TAMs.**

(A) Flow diagram of the two steps co-culturing system. (B and C) Western blotting analysis of indicated mesenchymal markers expression in GSCs co-culture with TAMs (GSCs educated HMC3).

**Supplementary Figure S14. Chemerin upregulates TAM infiltration and enhances mesenchymal features in GBM.**

(A, B, and D) Representative H&E staining images and area quantification of the maximum mouse brain cross-section of indicated chemerin overexpressed or knockdown orthotopic GBM tumors. GSC28, n = 3; GSC1, n = 4; GSC40, n = 3. Scale bars: 500 μm. (C) Survival curves of chemerin knockdown GSC40 orthotopic model. n = 6. (E and F) Western blotting analysis of indicated mesenchymal markers expression in chemerin overexpressed or knockdown GBM tumors. (G and H) Representative IHC images and staining quantification of TAMs infiltration in indicated GBM orthotopic tumors. n = 5. Scale bars: 50 μm. Data is presented as means ± SD. Differences in survival were analyzed using log-rank tests. Statistical significance in A, D, and G was analyzed with Student’s t test. Statistical significance in B was analyzed with paired *t* test. ^*^ P < 0.05, ^**^ P < 0.01.

**Supplementary Figure S15. NF-κB signaling pathway rather than AKT signaling pathway is responsible for chemerin’s mesenchymal promoting effect in GBM cells.**

(A) KEGG analysis showing top 10 pathway terms related to upregulated genes in *RARRES2* high-expression (> median value) patients in CGGA and TCGA GBM cohorts. (B) Western blotting analysis of time dependent AKT and MAPK signaling change in rChemerin treated GSCs. (C) Western blotting analysis of AKT signaling and indicated mesenchymal markers expression in chemerin overexpressed GSCs treated with MK2206.

**Supplementary Figure S16. GBM-derived chemerin activates NF-κB signaling in TAMs.**

(A and B) Western blotting analysis of indicated signaling pathways in HMC3 or THP-1 co-cultured with chemerin knockdown GSCs, treated with rChemerin or rTNF-α. (C and D) Western blotting analysis of indicated signaling pathways in HMC3 or THP-1 co-cultured with chemerin overexpressed GSCs, with CMKLR1 knockdown or treated with chemerin nAb or α-NETA.

**Supplementary Figure S17. Construction of chemerin overexpressed mGSCs**

Western blotting analysis and real-time PCR analysis of chemerin expression in chemerin overexpressed mGSCs. Data is presented as means ± SD. Statistical significance was analyzed with Student’s *t* test. ^****^ P < 0.0001.

**Supplementary Figure S18. Chemeirn and α-NETA can not influence the proliferation and apoptosis of GBM cells.**

(A and B) Proliferation and apoptosis analysis of indicated chemerin overexpressed GSCs treated with α-NETA. n = 3. Data is presented as means ± SD. Statistical significance was analyzed with one-way ANOVA.

**Supplementary Figure S19. Chemerin/CMKLR1 axis blockade decreases mesencymal features, NF-κB signaling activation, and TAMs infiltration in GBM tumors.**

(A) Representative IHC images and staining quantification of mesenchymal markers expression in indicated chemerin overexpressed orthotopic GBM tumors treated with α-NETA. n = 5. Scale bars: 50 μm. (B) Western blotting analysis of NF-κB signaling in indicated chemerin overexpressed GBM tumors treated with α-NETA. (C) Representative images of IHC and staining quantification of CCR2^+^ macrophages in indicated chemerin overexpressed orthotopic GBM tumors treated with α-NETA. n = 5. Data is presented as means ± SD. Statistical significance in A and C was analyzed with one-way ANOVA. ^*^ P < 0.05, ^**^ P < 0.01, ^***^ P < 0.001.

**Supplementary Figure S20. Human GSCs-derived chemeirn has similar effects in the infiltration and polarization of both mouse BMDM and human TAMs.**

(A) *In vitro* migration assay of mBMDM co-cultured with chemerin knockdown or overexpressed GSCs, with indicated treatment. n = 3. (B) Real-time PCR analysis of the inflammatory factors (IL-1β and TNF-α) and immunosuppressive factors (TGF-β and CD163) expression in BMDM co-cultured with chemerin overexpressed GSCs, treated with chemerin nAb or α-NETA. n=3. Data is presented as means ± SD. Statistical significance was analyzed using one-way ANOVA. ns = not significant, ^*^ P < 0.05, ^**^ P < 0.01, ^***^ P < 0.001, ^****^ P < 0.0001.

**Supplementary Figure S21. Chemeirn/CMKLR1 axis blockade improves anti-tumor functions of T cells in GBM.**

(A) Flow cytometry analysis of the proportion of CD3^+^, CD4^+^, and CD8^+^ T cell populations. (B-D) Flow cytometric analysis of PD-1 (B), TNF-α (C), and IFN-γ (D) in indicated T cell populations from mGSC orthotopic tumors. n = 5. (E and F) Flow cytometry analysis of immune cell populations and M1/M2 polarization of CD11b^+^ F4/80^+^ monocyte/macrophage in peripheral system of mGSC orthotopic model. n = 3. Data is presented as means ± SD. Statistical significance in E and F was analyzed with Student’s *t* test. Statistical significance in A to D was analyzed with one-way ANOVA. ns = not significant, ^*^ P < 0.05, ^**^ P < 0.01.

**Supplementary Table legends**

**Supplementary Table S1**

a: Samples used for bioinformatic analysis in CGGA dataset.

b: Samples used for bioinformatic analysis in TCGA dataset.

c: Samples used for bioinformatic analysis in Rembrandt dataset.

d: Samples used for bioinformatic analysis in Gravendeel dataset.

**Supplementary Table S2**

Six gene sets from the MSigDB database involved in cellular mesenchymal transition.

**Supplementary Table S3**

a: Differential expression genes between mesenchymal subtype GBM and proneural GBM in TCGA.

b: Genes correlated with TAM score in TCGA.

c: Differential expression genes between mesenchymal subtype GBM and proneural GBM in CGGA.

d: Genes correlated with TAM score in CGGA.

e: Differential expression genes between mesenchymal subtype GBM and proneural GBM in Gravendeel.

f: Genes correlated with TAM score in Gravendeel.

g: Intersection of differential expression genes and correlation genes in three datasets.

h: Intersection of genes having prognostic value in GBM.

i: Protein-protein interaction and co-expression analysis of three selected genes.

**Supplementary Table S4**

Association between RARRES2 high/low expression subgroups and clinicopathologic variables in TCGA and CGGA GBM.

**Supplementary Table S5**

Lipid metabolism related GSVA scores in GBM cells.

**Supplementary Table S6**

Association between CMKLR1/RARRES2 modules and clinicopathologic variables in TCGA GBM.

**Supplementary Table S7**

Sequencing information of siRNAs and primers.
