# Supplementary figures and images for: Chemerin enhances mesenchymal features of glioblastoma by establishing autocrine and paracrine networks in a CMKLR1-dependent manner
Source: Oncogene. 2022 Apr 22;41(21):3024–36. doi: 10.1038/s41388-022-02295-w (PMC9122825; doi:10.1038/s41388-022-02295-w)

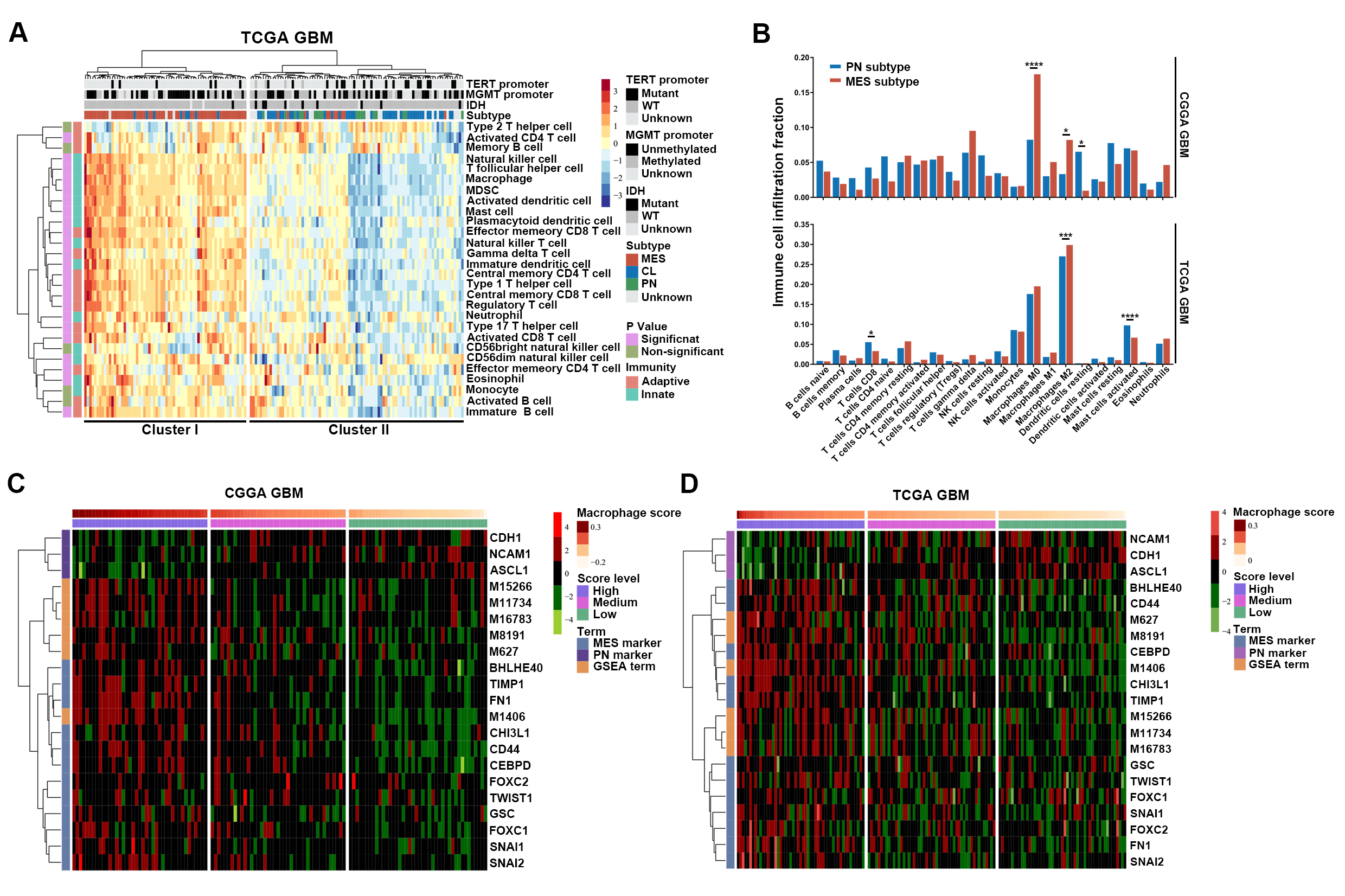

Supplement: Supplementary file 2 — Supplementary Figure S1 [file 41388_2022_2295_MOESM2_ESM.tif]

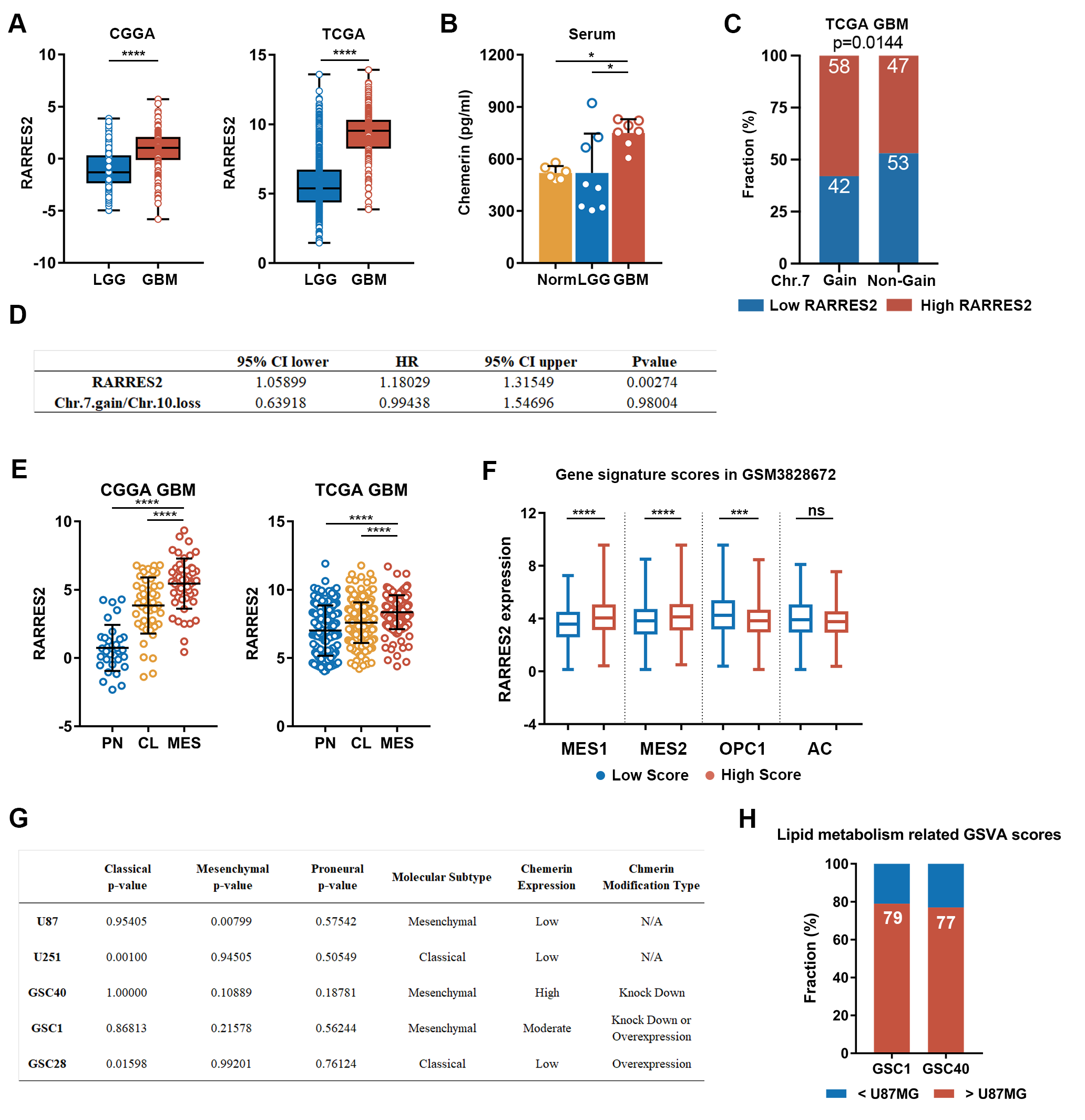

Supplement: Supplementary file 3 — Supplementary Figure S2 [file 41388_2022_2295_MOESM3_ESM.tif]

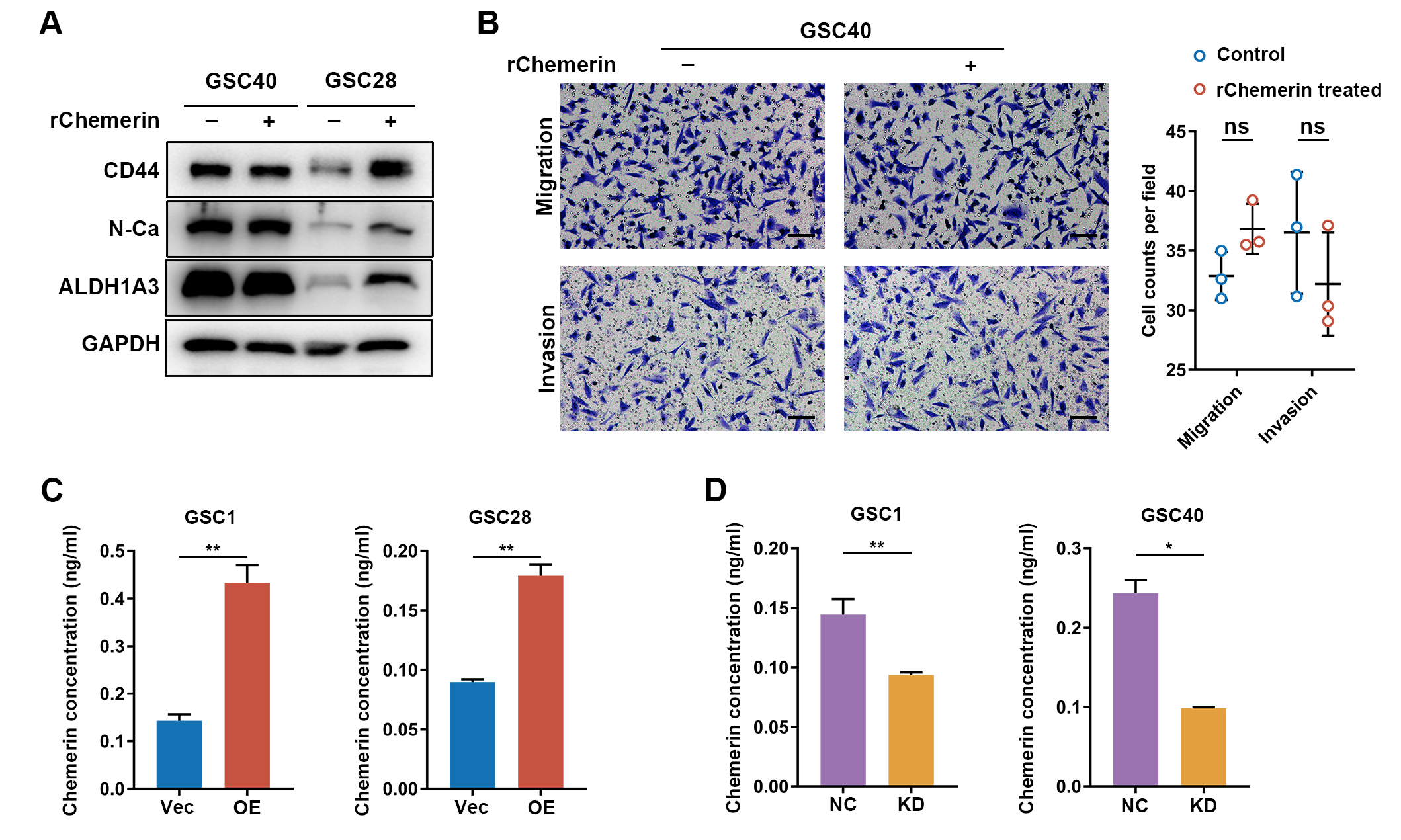

Supplement: Supplementary file 4 — Supplementary Figure S3 [file 41388_2022_2295_MOESM4_ESM.tif]

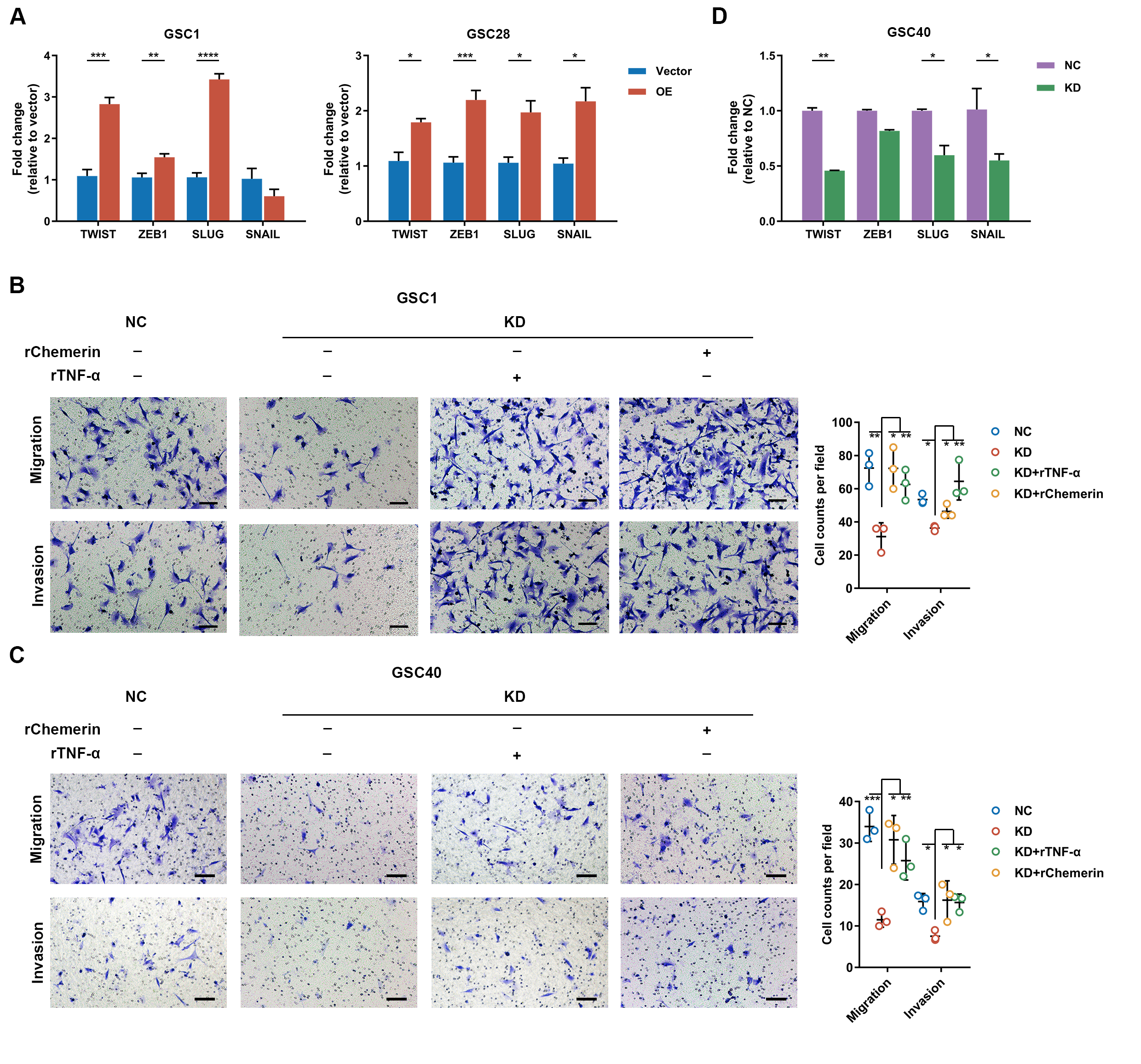

Supplement: Supplementary file 5 — Supplementary Figure S4 [file 41388_2022_2295_MOESM5_ESM.tif]

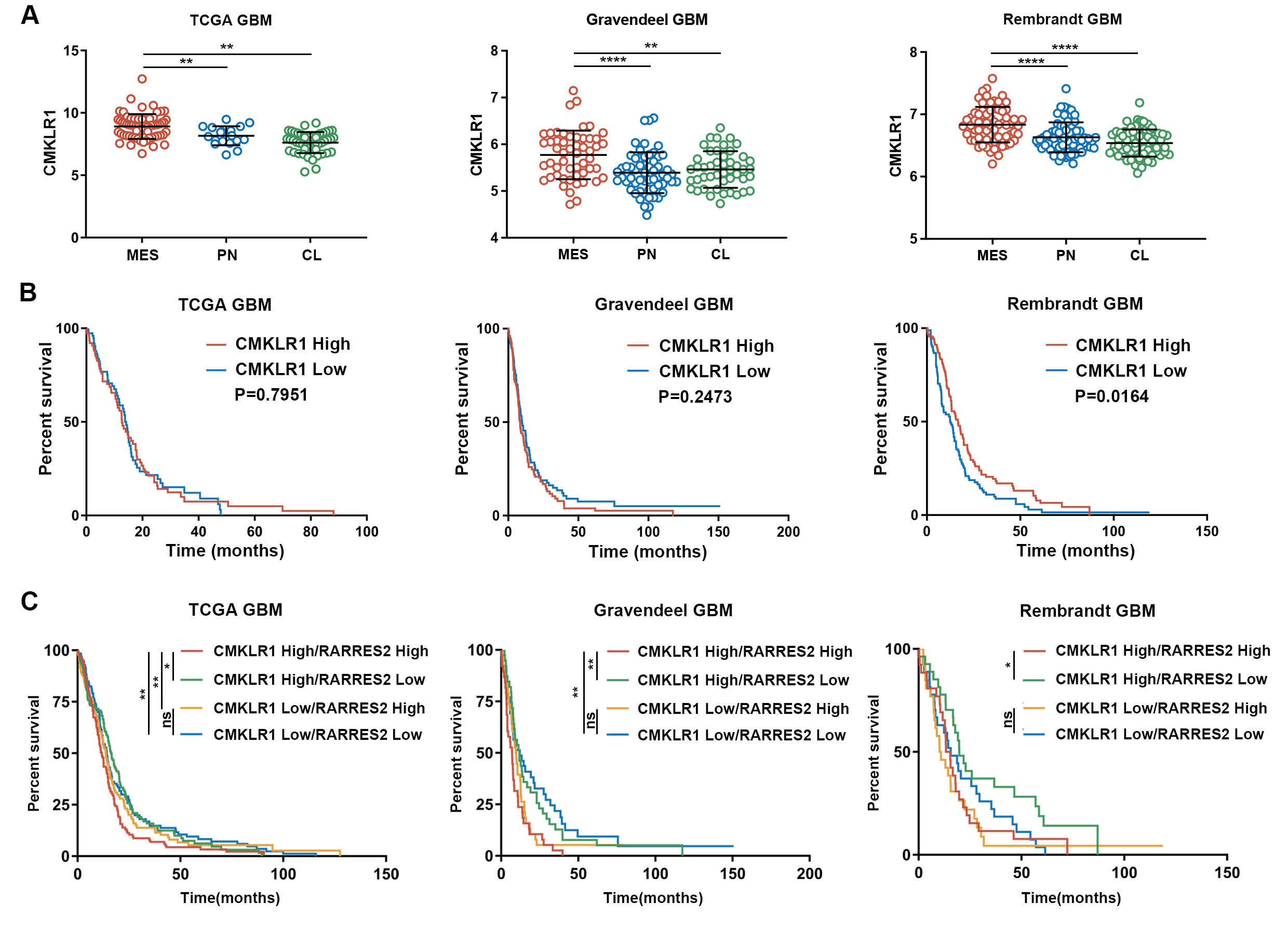

Supplement: Supplementary file 6 — Supplementary Figure S5 [file 41388_2022_2295_MOESM6_ESM.tif]

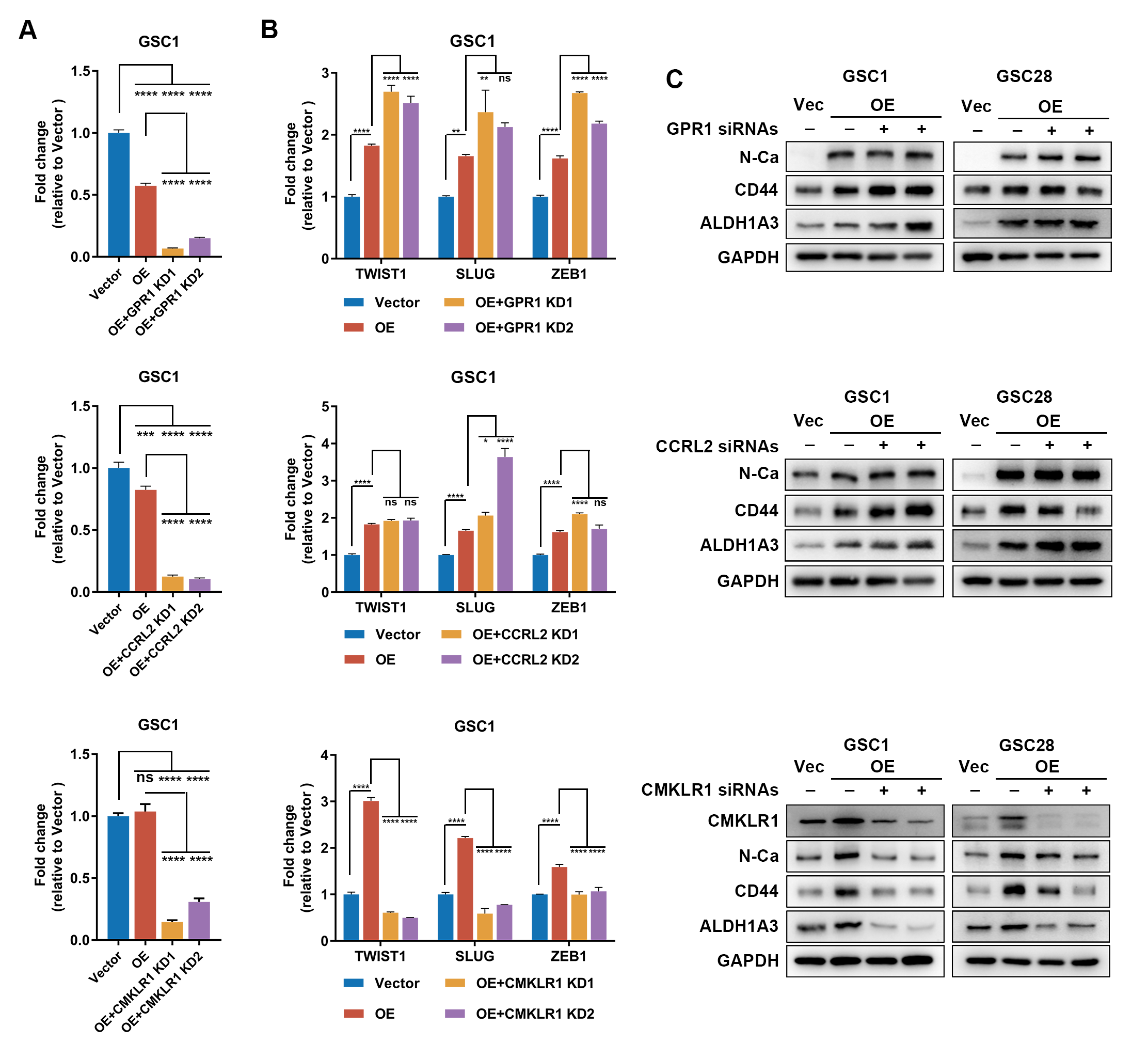

Supplement: Supplementary file 7 — Supplementary Figure S6 [file 41388_2022_2295_MOESM7_ESM.tif]

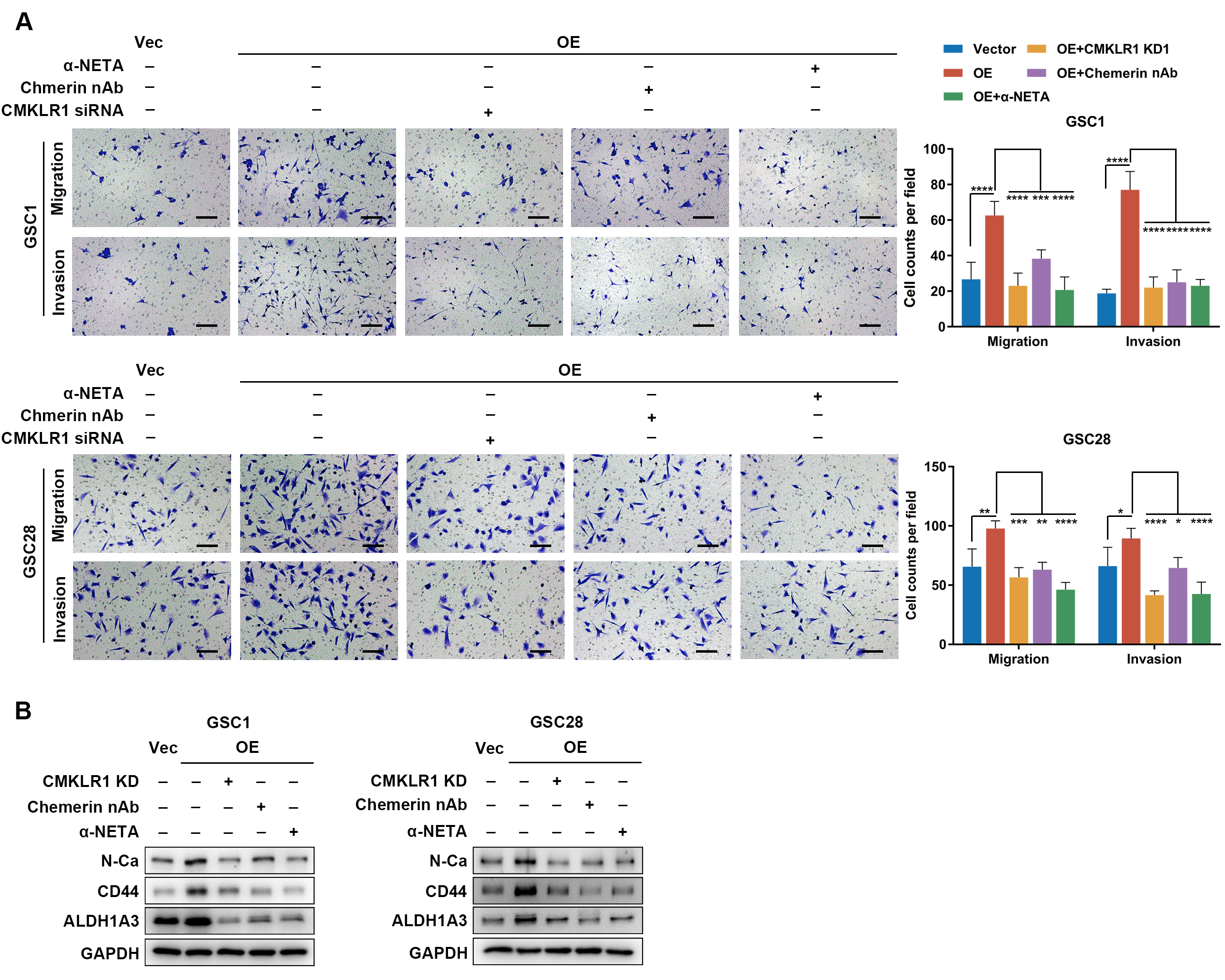

Supplement: Supplementary file 8 — Supplementary Figure S7 [file 41388_2022_2295_MOESM8_ESM.tif]

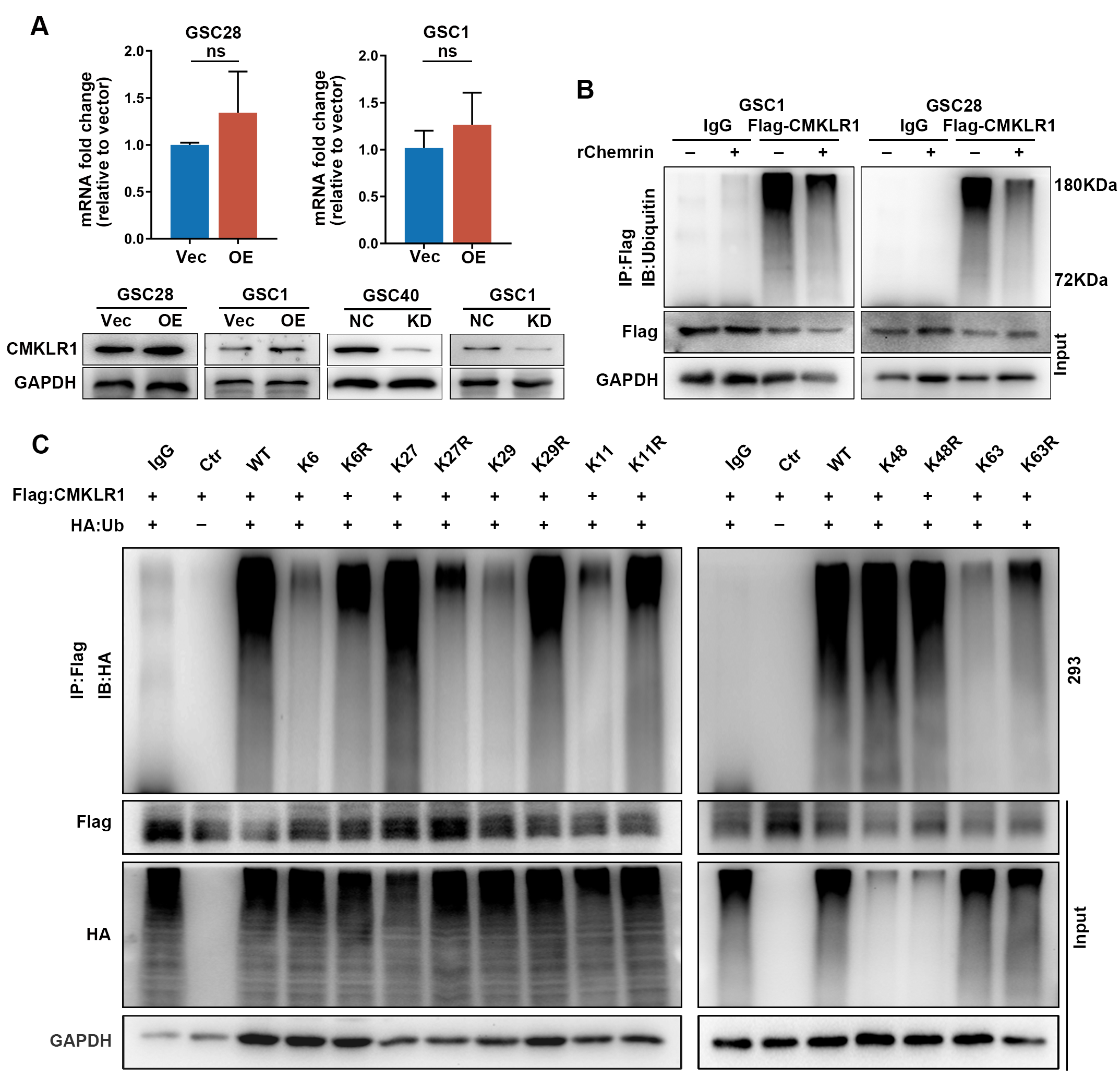

Supplement: Supplementary file 9 — Supplementary Figure S8 [file 41388_2022_2295_MOESM9_ESM.tif]

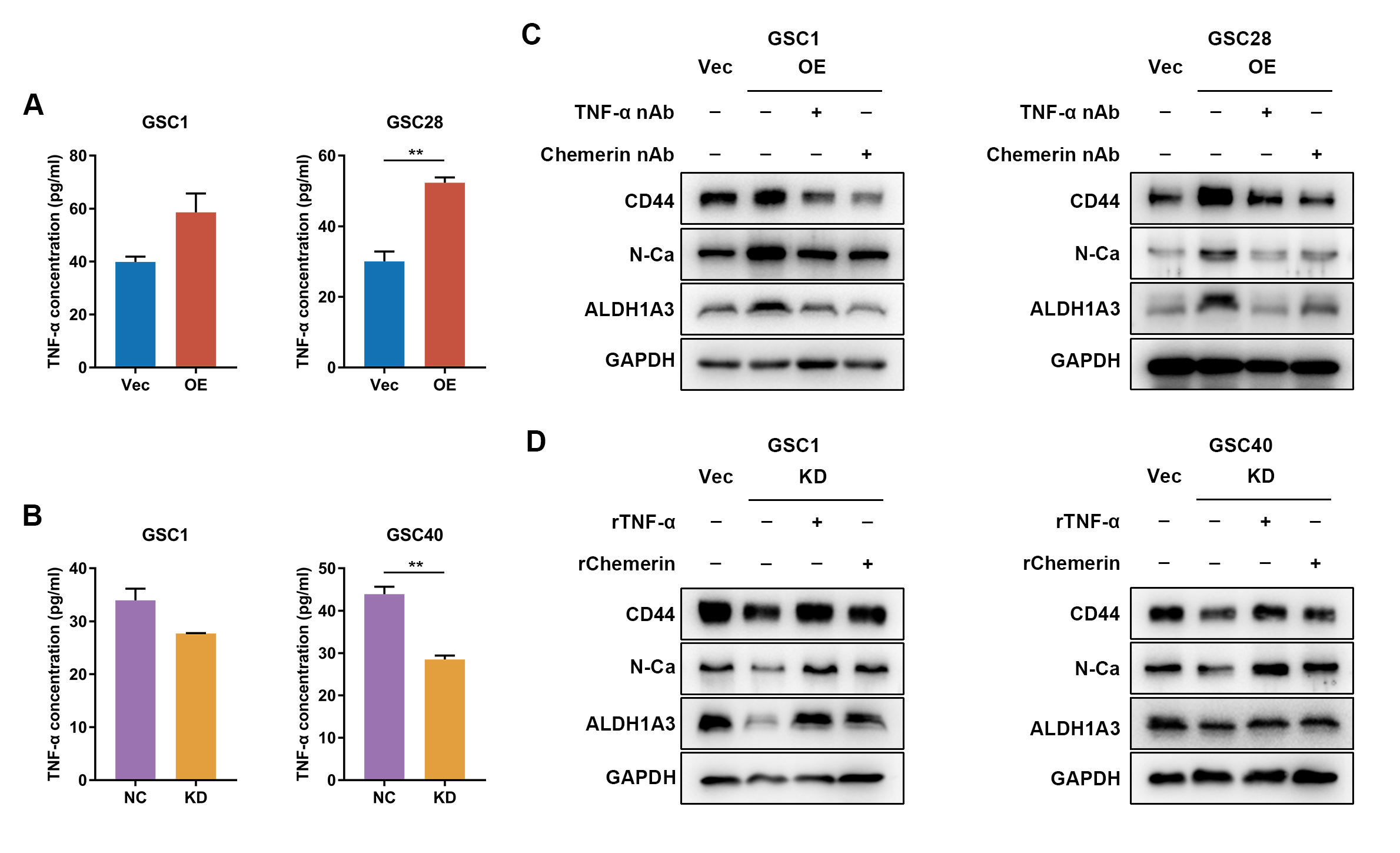

Supplement: Supplementary file 10 — Supplementary Figure S9 [file 41388_2022_2295_MOESM10_ESM.tif]

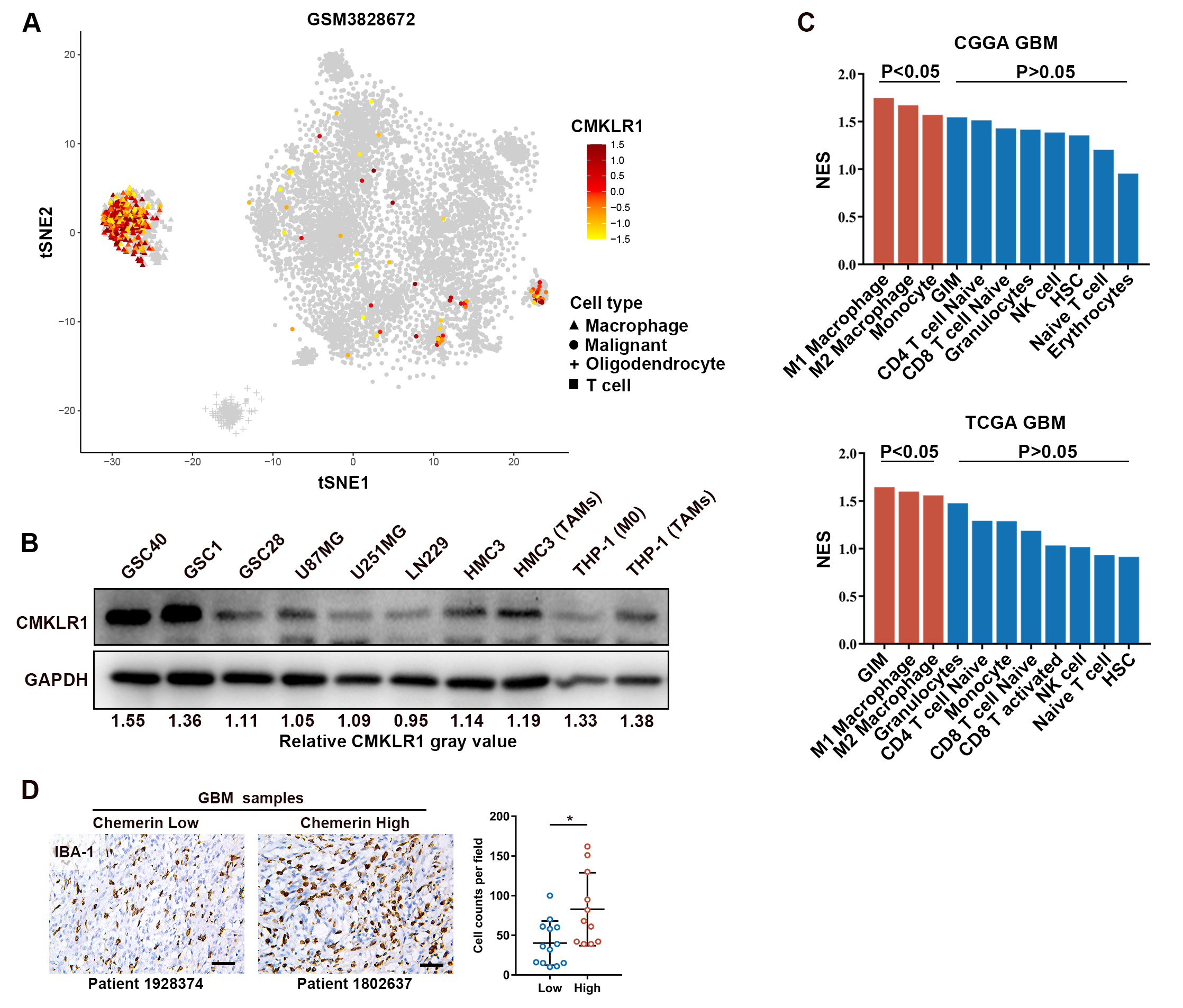

Supplement: Supplementary file 11 — Supplementary Figure S10 [file 41388_2022_2295_MOESM11_ESM.tif]

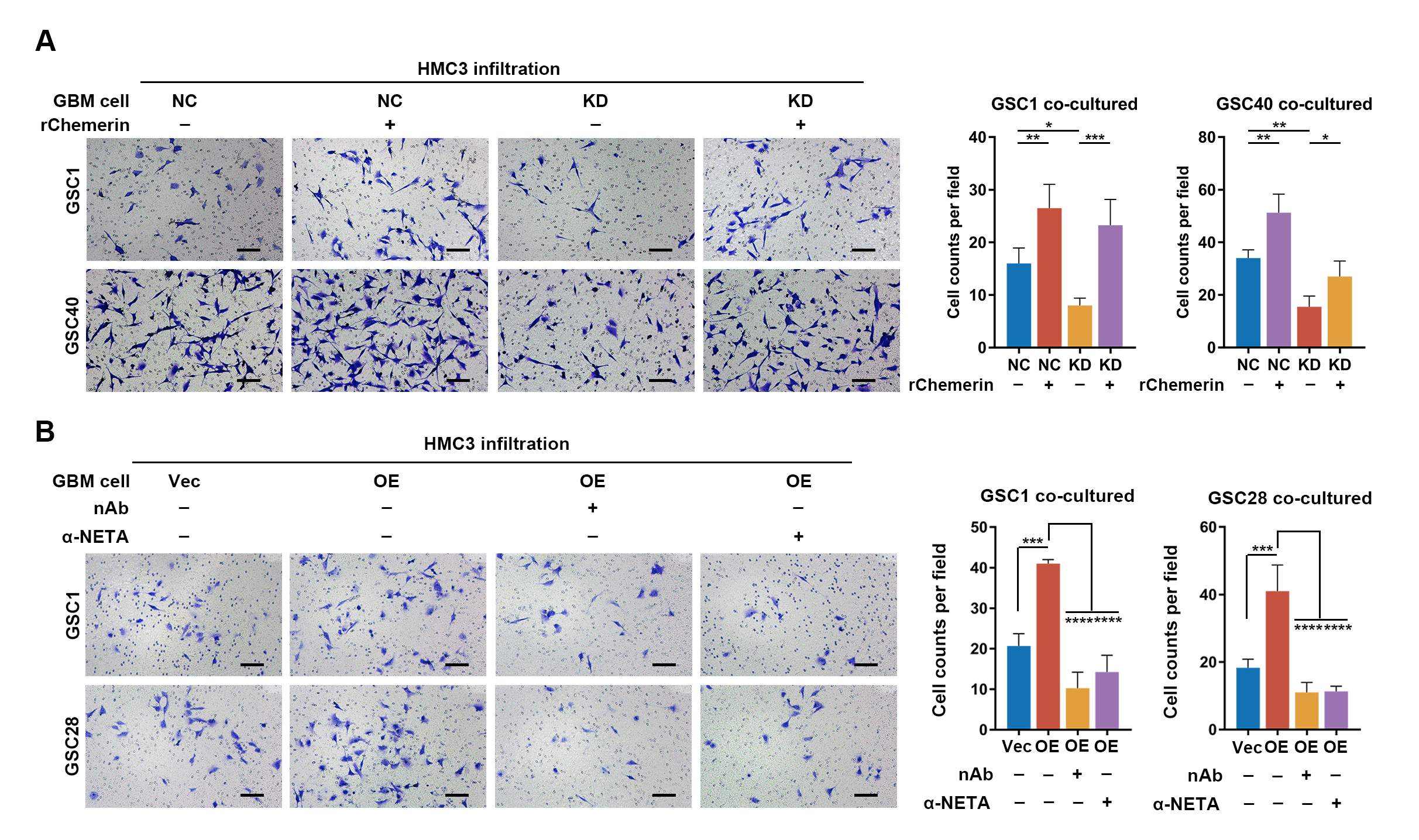

Supplement: Supplementary file 12 — Supplementary Figure S11 [file 41388_2022_2295_MOESM12_ESM.tif]

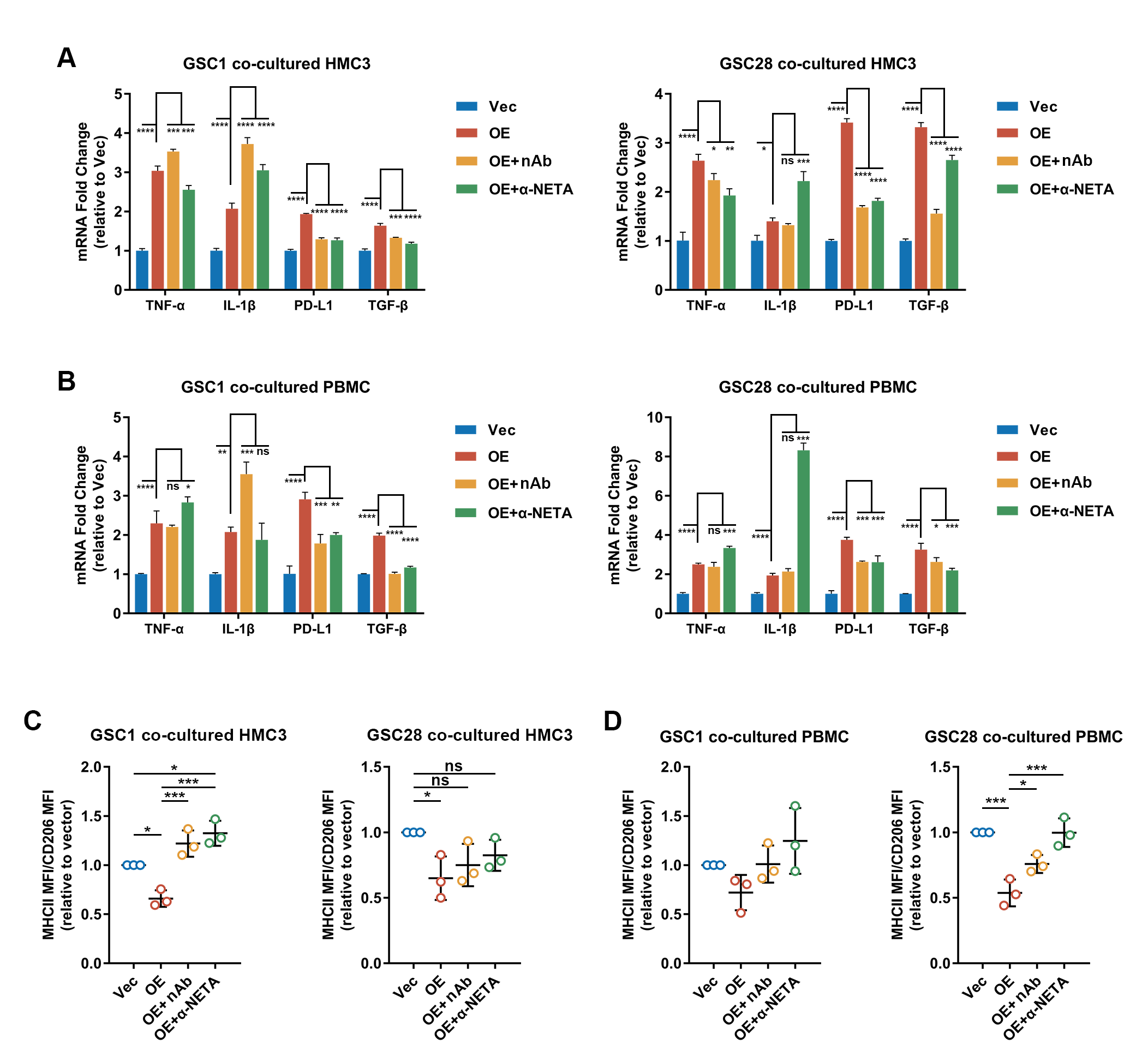

Supplement: Supplementary file 13 — Supplementary Figure S12 [file 41388_2022_2295_MOESM13_ESM.tif]

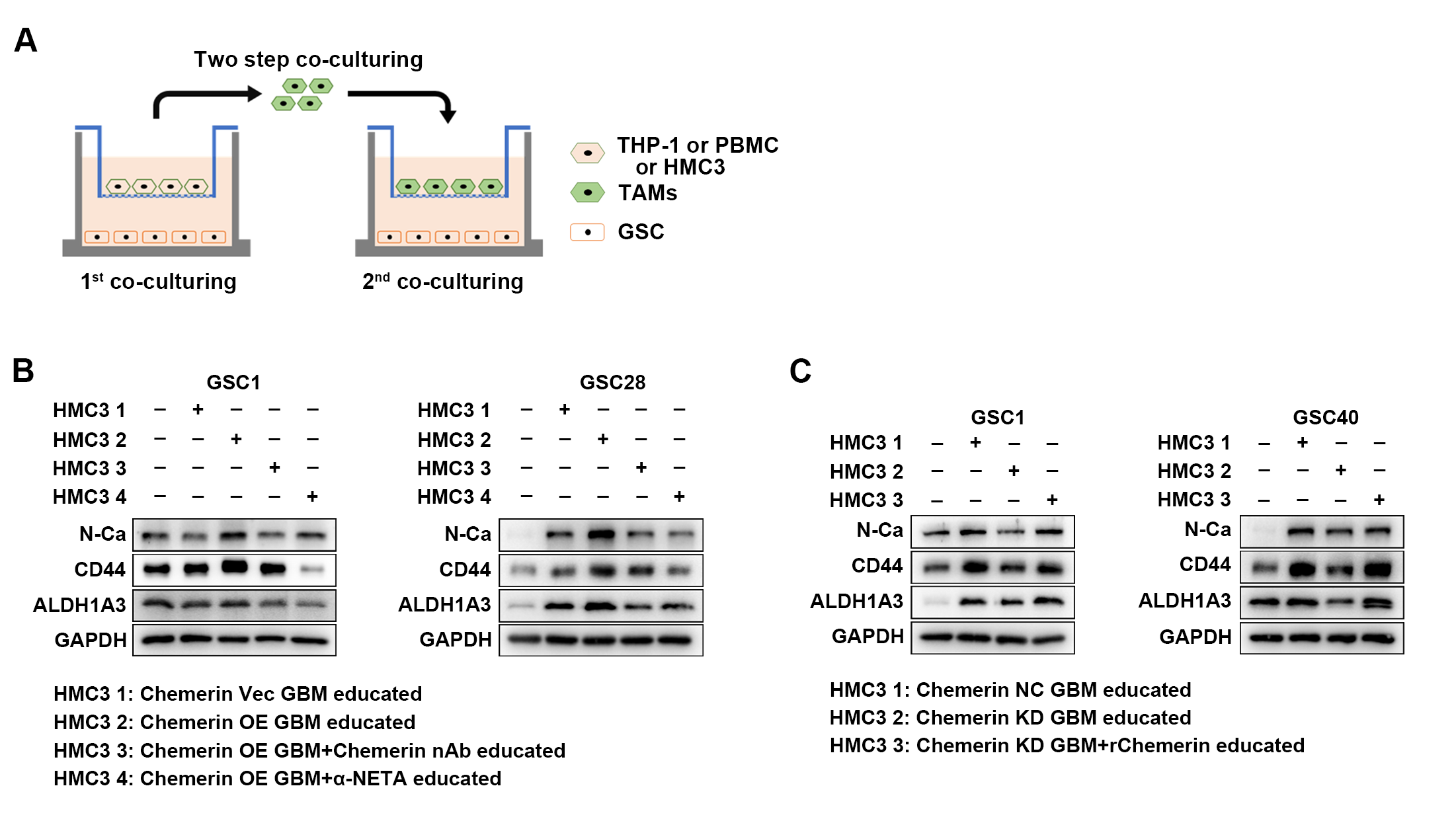

Supplement: Supplementary file 14 — Supplementary Figure S13 [file 41388_2022_2295_MOESM14_ESM.tif]

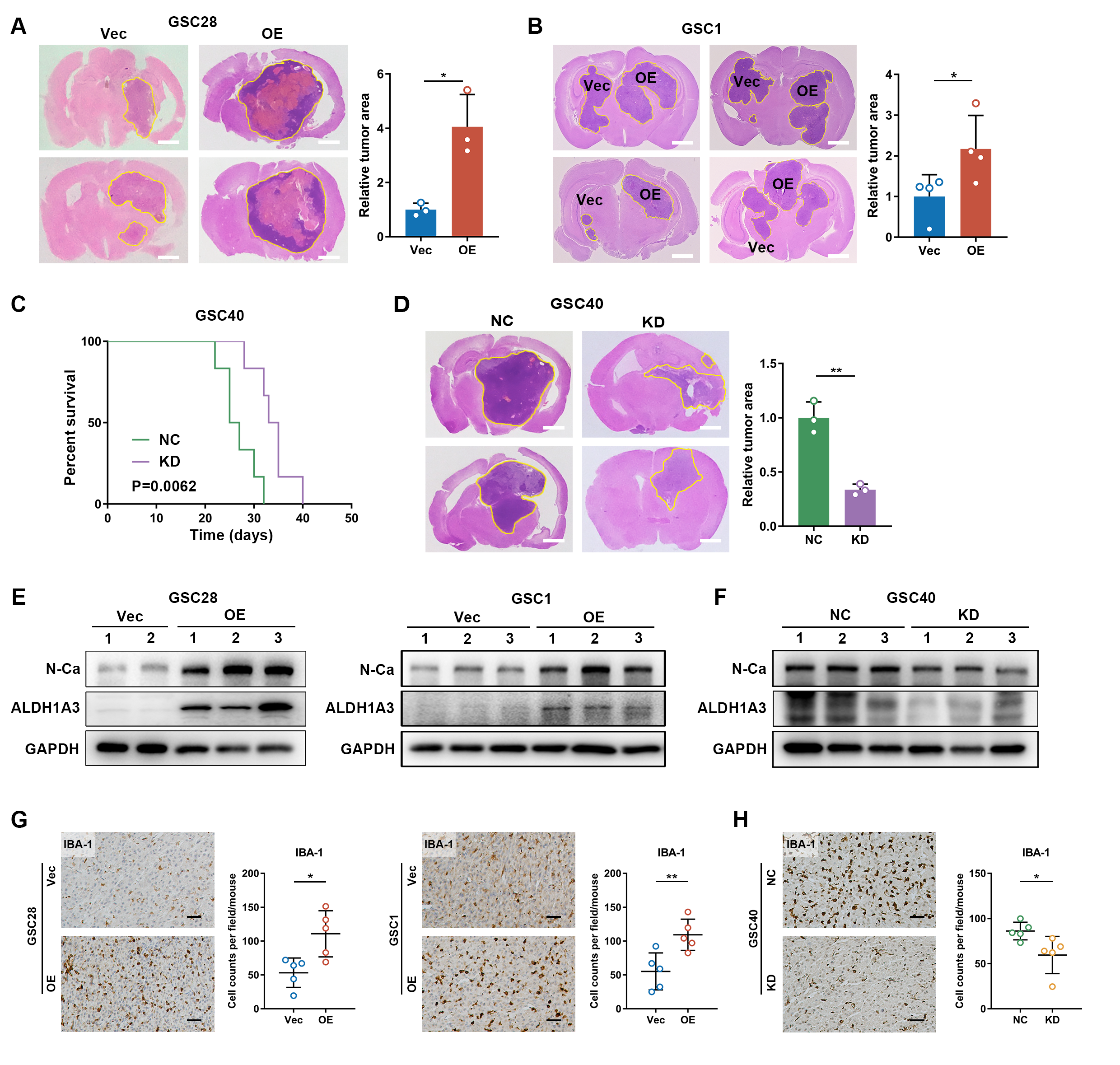

Supplement: Supplementary file 15 — Supplementary Figure S14 [file 41388_2022_2295_MOESM15_ESM.tif]

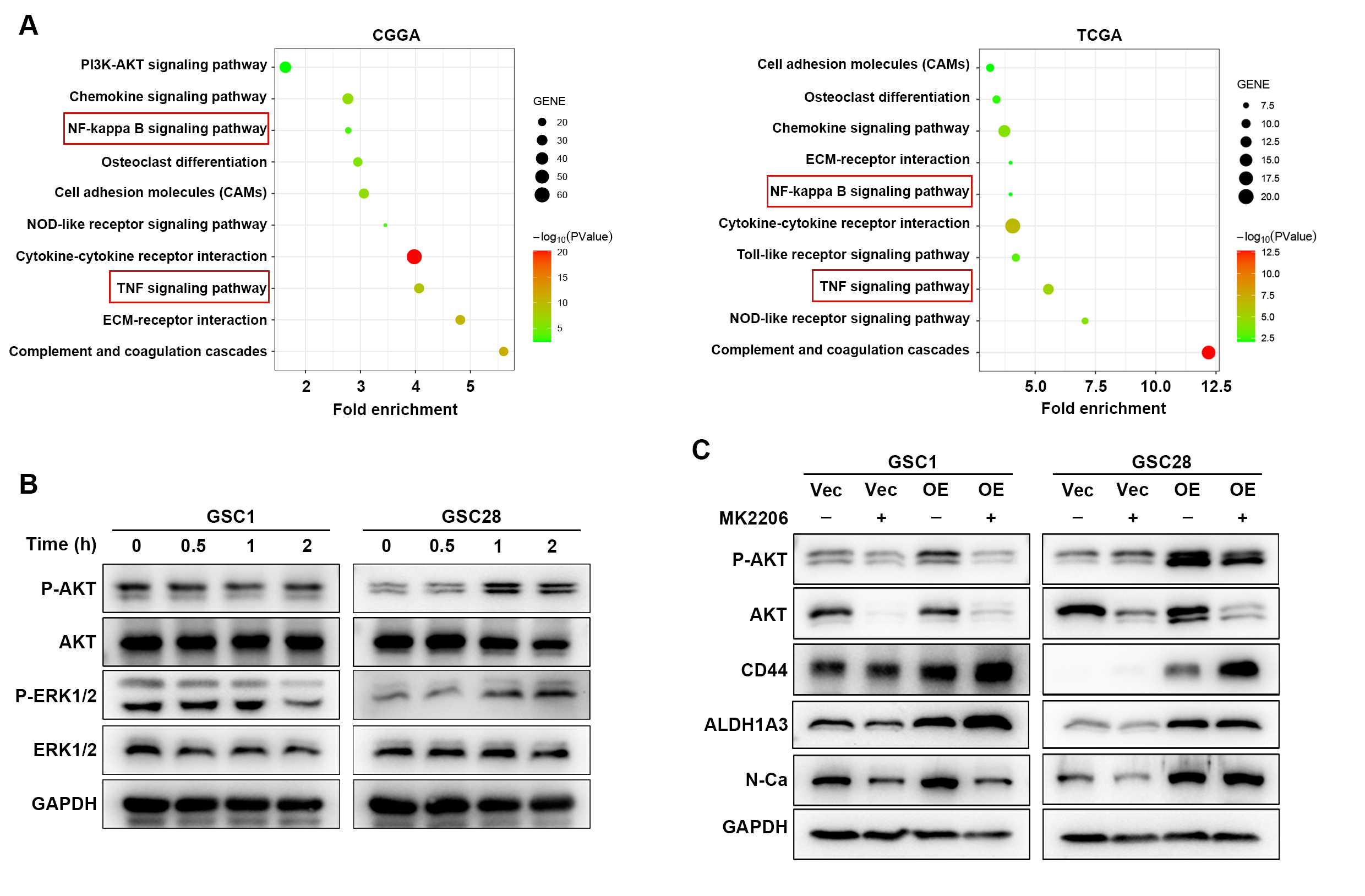

Supplement: Supplementary file 16 — Supplementary Figure S15 [file 41388_2022_2295_MOESM16_ESM.tif]

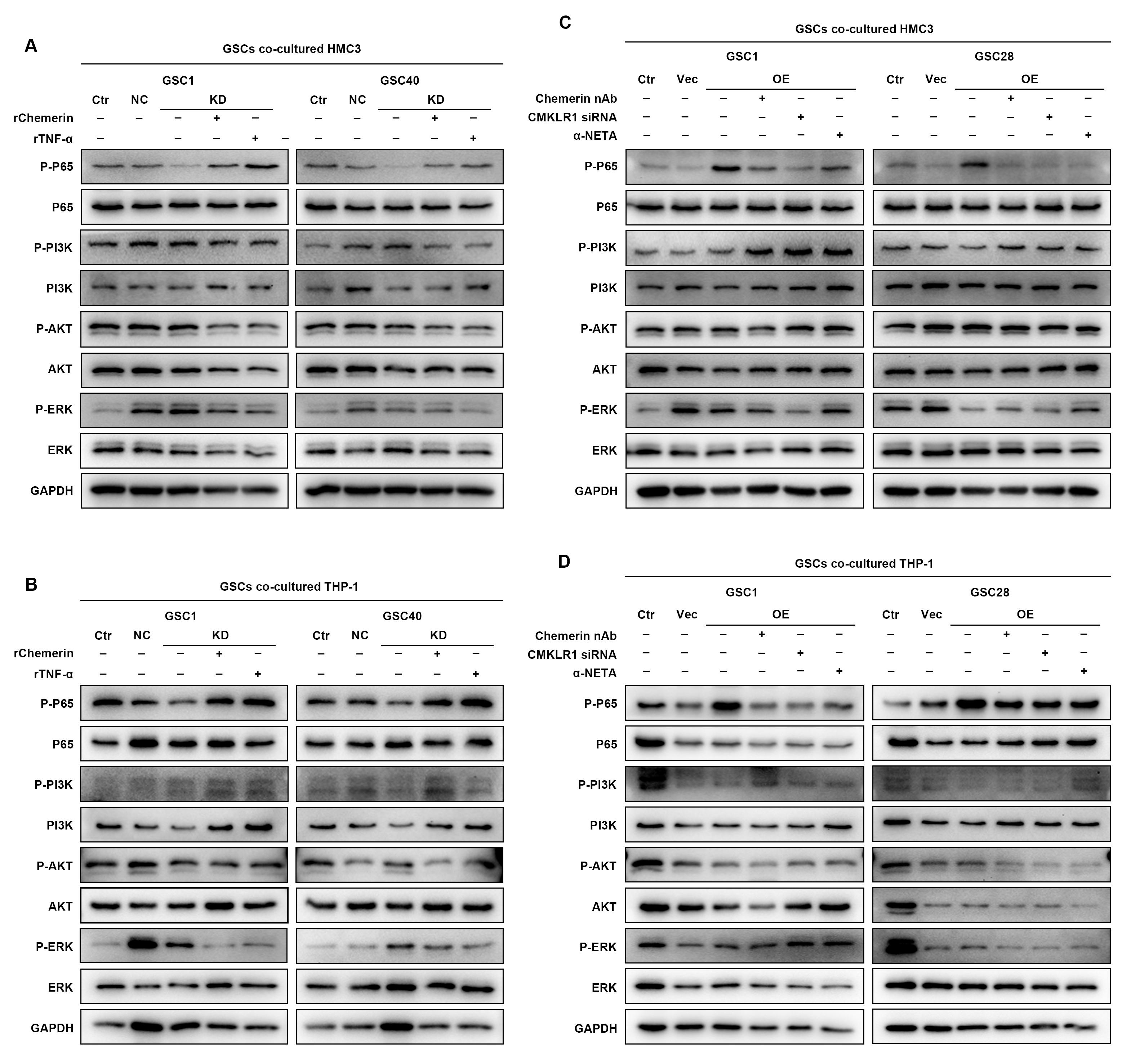

Supplement: Supplementary file 17 — Supplementary Figure S16 [file 41388_2022_2295_MOESM17_ESM.tif]

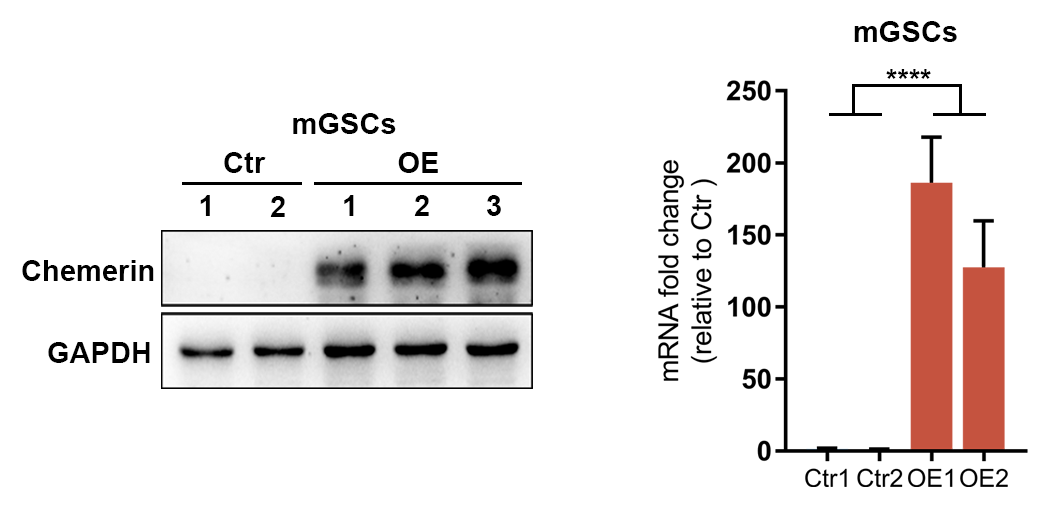

Supplement: Supplementary file 18 — Supplementary Figure S17 [file 41388_2022_2295_MOESM18_ESM.tif]

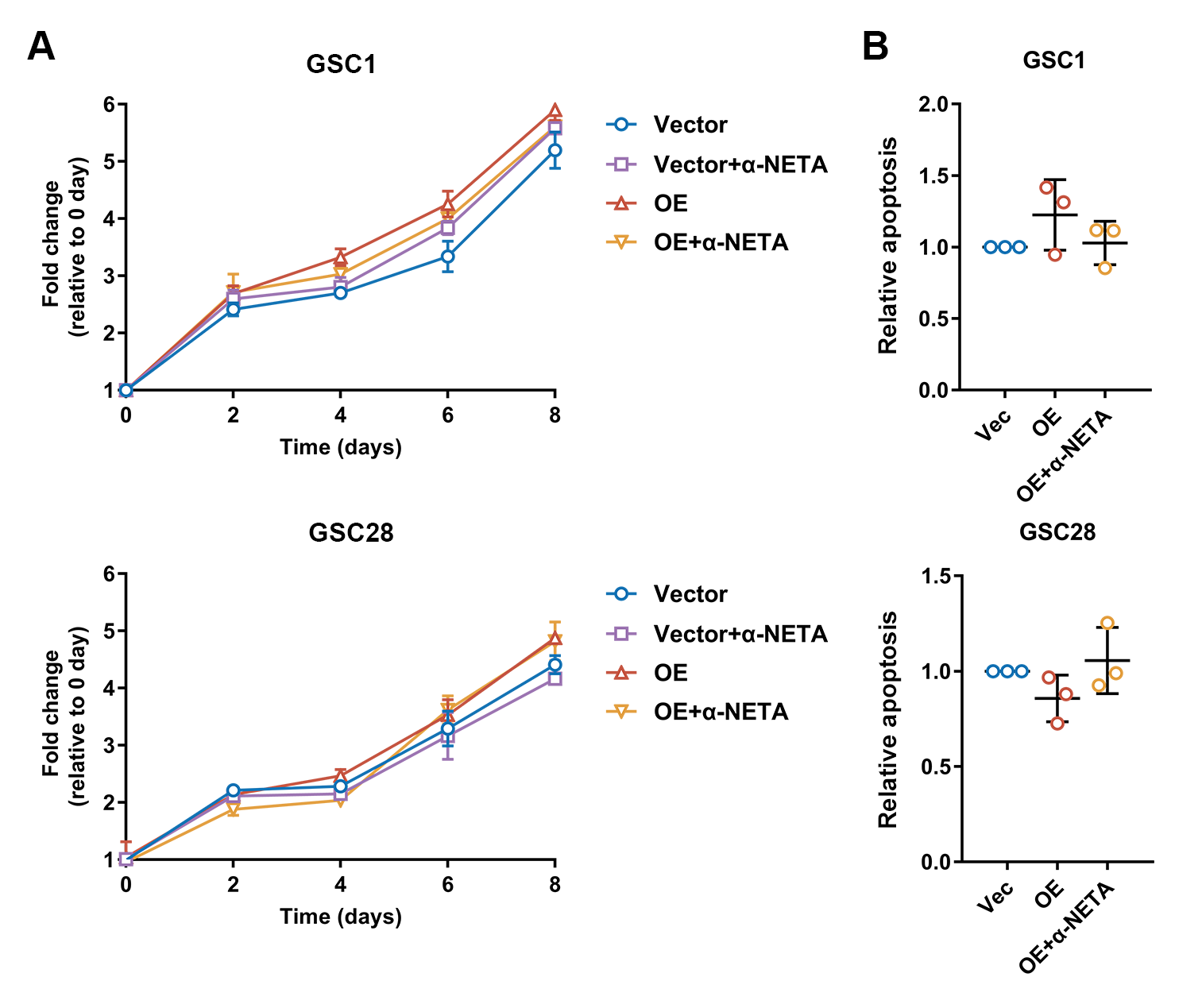

Supplement: Supplementary file 19 — Supplementary Figure S18 [file 41388_2022_2295_MOESM19_ESM.tif]

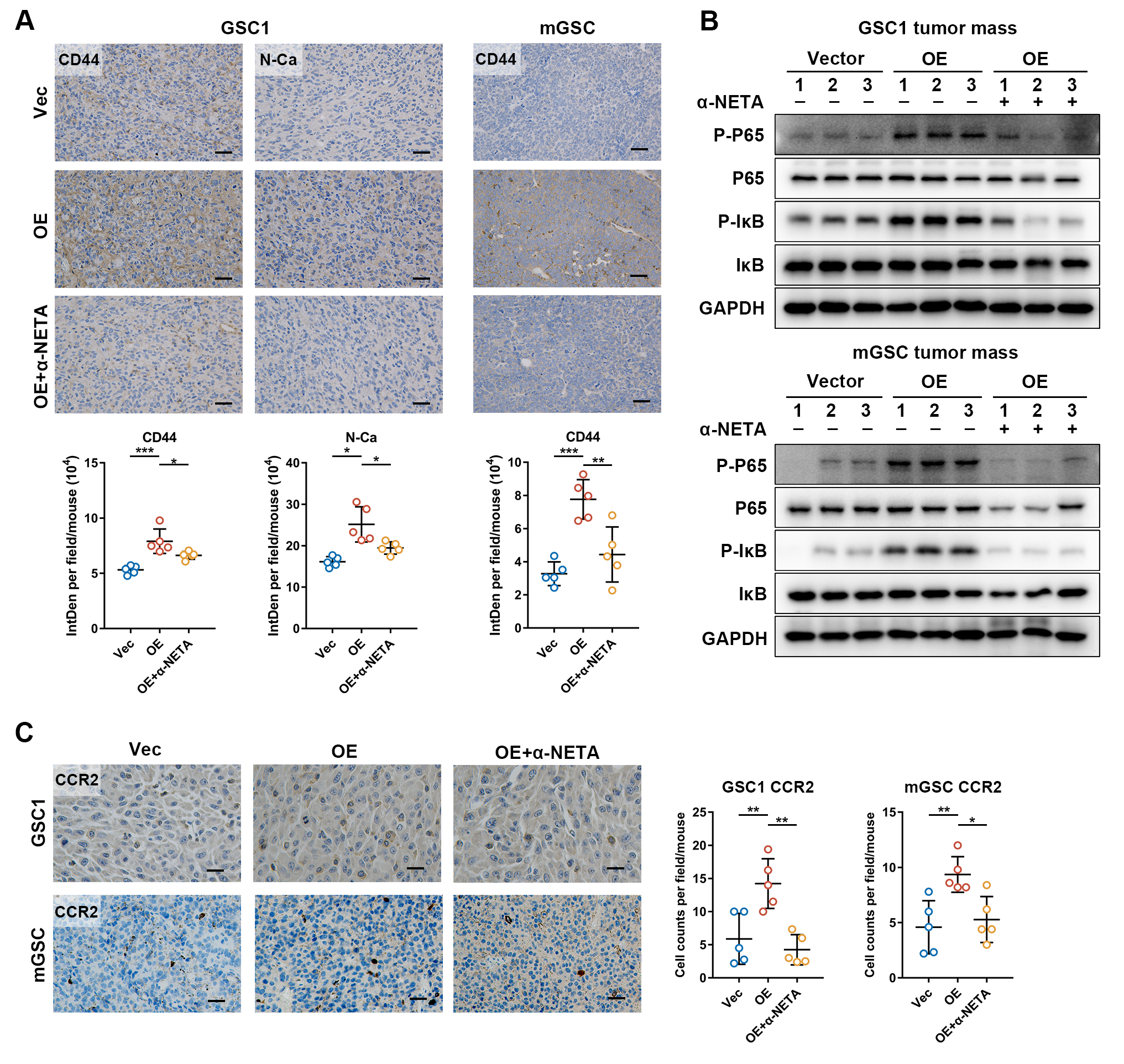

Supplement: Supplementary file 20 — Supplementary Figure S19 [file 41388_2022_2295_MOESM20_ESM.tif]

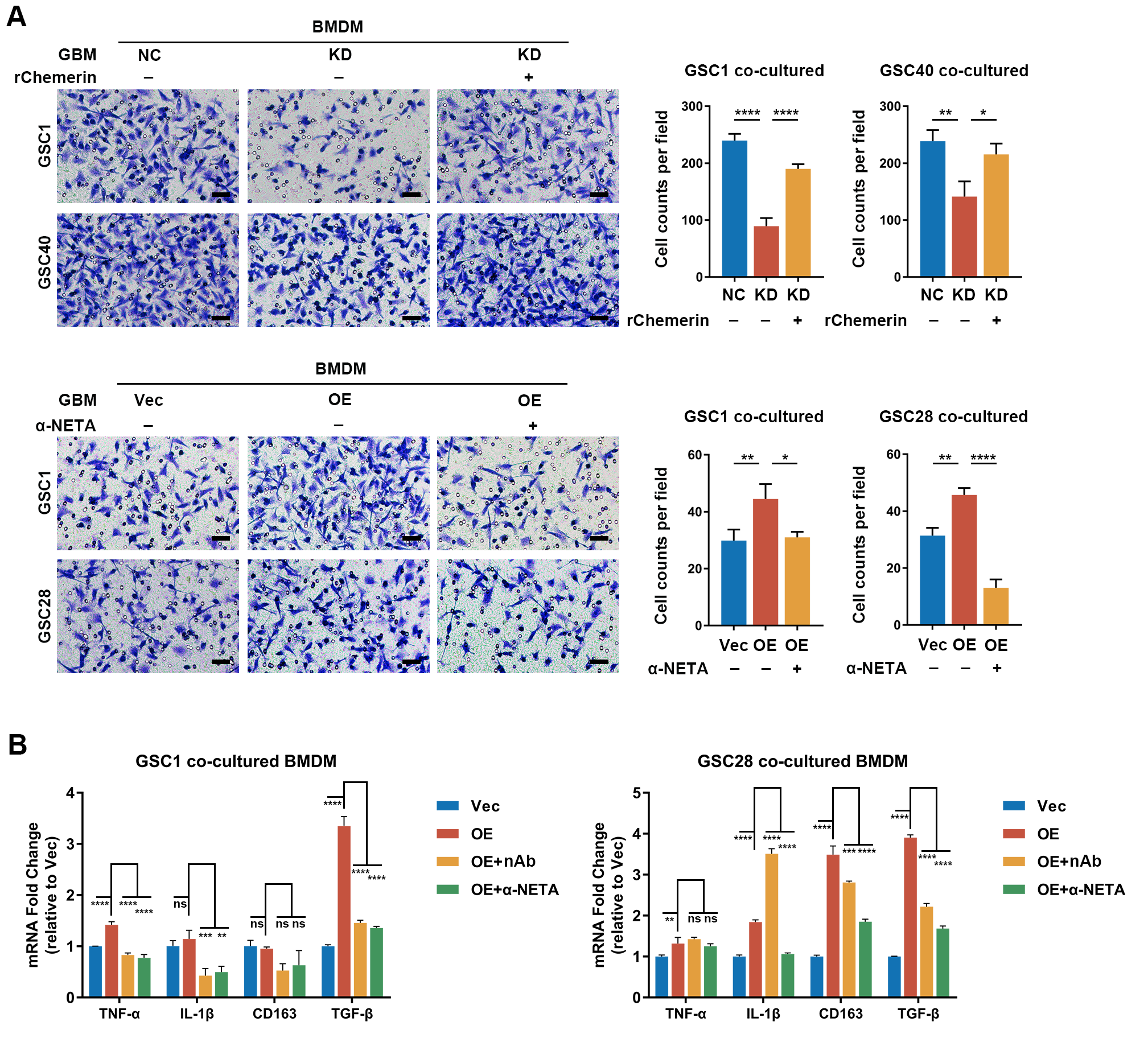

Supplement: Supplementary file 21 — Supplementary Figure S20 [file 41388_2022_2295_MOESM21_ESM.tif]

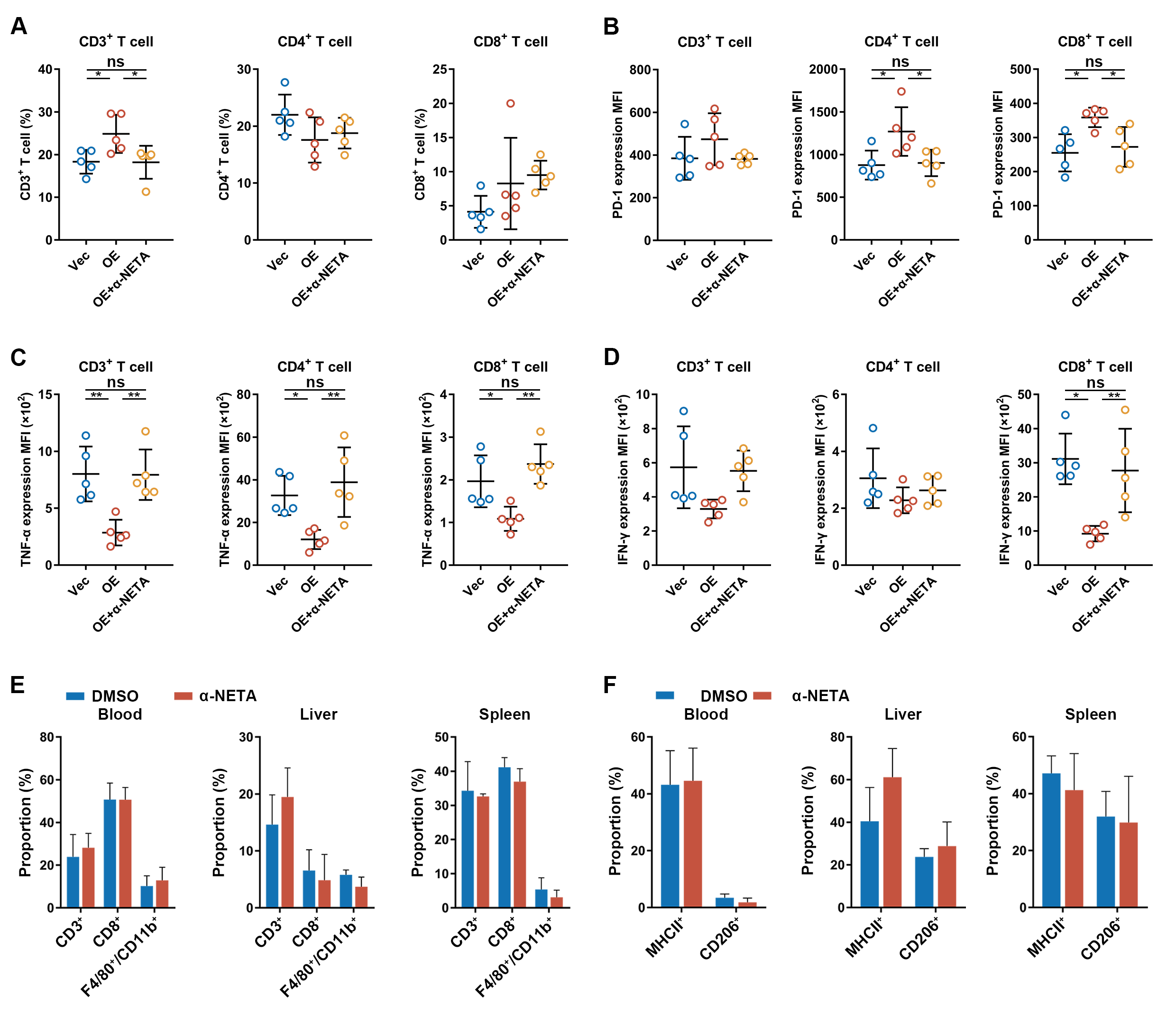

Supplement: Supplementary file 22 — Supplementary Figure S21 [file 41388_2022_2295_MOESM22_ESM.tif]
